# Controlled Growth of Rare-Earth-Doped TiO_2_ Thin Films on III–V Semiconductors for Hybrid Quantum Photonic Interfaces

**DOI:** 10.1021/acsaom.5c00585

**Published:** 2026-02-04

**Authors:** Henry C. Hammer, Caleb Whittier, Nathan A. Helvy, Christopher Rouleau, Nabil D. Bassim, Ravitej Uppu

**Affiliations:** † Department of Physics and Astronomy, 4083 The University of Iowa, Iowa City, Iowa 52242, United States; ‡ Department of Materials Science and Engineering, 3710 McMaster University, Hamilton, Ontario L8S 4L7, Canada; § Center for Nanophase Materials Sciences, 6146 Oak Ridge National Laboratory, Oak Ridge, Tennessee 37831, United States; ∥ Canadian Centre for Electron Microscopy, McMaster University, Hamilton, Ontario L8S 4M1, Canada

**Keywords:** rare-earth spins, optical coherence, quantum photonics, quantum information, heteroepitaxy

## Abstract

Quantum photonic networks require two distinct functionalities: bright single-photon sources and long-lived quantum memories. III–V semiconductor quantum dots (QDs) excel as deterministic and coherent photon emitters, while rare-earth ions such as erbium (Er^3+^) in crystalline oxides offer exceptional spin and optical coherence at telecom wavelengths. Combining these systems and their functionalities via direct epitaxy is challenging due to lattice mismatch and incompatible growth conditions. Here, we demonstrate the low-temperature pulsed laser deposition of Er^3+^-doped TiO_2_ thin films directly on GaAs and GaSb substrates. Controlled surface preparation with an arsenic cap and an oxygen-deficient buffer layer enables the growth of epitaxial anatase TiO_2_ (001) at ∼390 °C with sub-300 pm surface roughness, while avoiding interface degradation. In contrast, high-temperature oxide desorption or growth temperatures drive the transition to a rough, polycrystalline rutile film, as confirmed by transmission electron microscopy. Minimal coincident interface area (MCIA) modeling explains the orientation-selective growth on GaAs and GaSb. Raman and cryogenic photoluminescence excitation spectroscopy verify the crystal phase and optical activation of Er^3+^ ions. This multiparameter growth strategy helps preserve III–V quantum dot functionality and yields smooth surfaces suitable for low-loss nanophotonic structures. Our results establish a materials platform for monolithically integrating rare-earth quantum memories with semiconductor photon sources, paving the way toward scalable hybrid quantum photonic chips.

## Graphical abstract

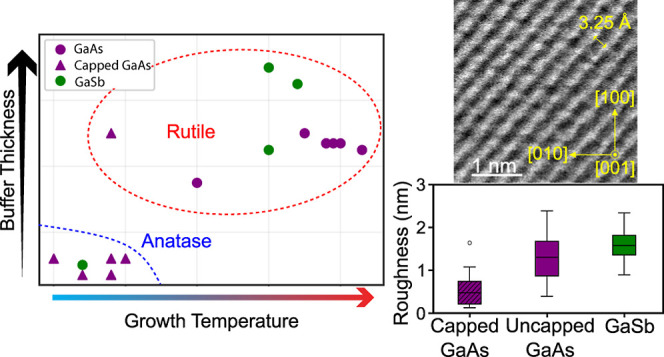

## 1 Introduction

Quantum photonic networks require material platforms that support both bright, deterministic photon sources^1,2^ and long-lived quantum memories,^3,4^ ideally operating at telecommunication wavelengths.^5,6^ These two functionalities impose fundamentally different material requirements.^7^ While epitaxial III–V quantum dots (QDs) offer bright and coherent photon emission,^8,9^ rare-earth ions (REIs) in crystalline oxide hosts provide exceptionally long (millisecond-scale) spin and optical coherence times, making them well suited as quantum memories.^10–12^ Combining these complementary systems into a monolithic architecture would enable the development of recently proposed hybrid quantum repeater chains^13^ for next-generation quantum networks. However, the growth of such a heterogeneous photonic interface remains challenging due to lattice mismatches, thermal processing, and growth chemistries.

Heterogeneous integration methods, such as flip-chip bonding, have enabled functional integration of III–V or silicon nanophotonics (e.g., waveguides and cavities) to REI-doped crystals.^14–19^ However, these methods suffer from alignment complexity, uncontrolled interfaces, and bonding-induced losses that degrade coherence.^14^ In contrast, monolithic integration via direct growth enables atomic-scale control of the REI-semiconductor interface while eliminating bonding layers and improving alignment tolerances. This approach supports scalable hybrid photonic integrated circuits in which spin qubits and photon sources share the same optical mode, a key requirement for efficient spin-photon entanglement and low-loss quantum state transfer.^13,20^ Direct growth of erbium-doped oxide thin films on silicon, including TiO_2_,^21–24^ CeO_2_,^25,26^ and Y_2_O_3_,^27–29^ have shown narrow inhomogeneous optical line widths, including spin coherence time (*T*_2_) exceeding 400 μs relevant for scalable quantum memories. Yet, because silicon lacks efficient single-photon sources, these platforms require extreme Purcell enhancement of the REI’s optical lifetimes to approach the brightness of III–V QDs.

A more versatile strategy is to integrate REI-doped oxides directly with III–V semiconductors. Among potential oxide hosts, TiO_2_ is especially promising for Er^3+^-based quantum memories.^30,31^ It combines a wide bandgap and high refractive index at telecom wavelengths with a nearly nuclear-spin-free lattice environment, while supporting dry-etching processes compatible with III–V nanofabrication.^32–34^ Er^3+^ ions substitute at the Ti^4+^ sites of nonpolar *D*_2h_/*D*_2d_ symmetry in rutile/anatase phases, which suppresses permanent dipole formation and supports long optical lifetimes and narrow line widths, recently measured in both bulk and thin-film TiO_2_ on silicon.^23,35–37^ Despite its promise, direct growth of Er^3+^/TiO_2_ on III–V semiconductors for quantum photonics has remained largely unexplored. Previous pulsed laser deposition (PLD) studies of TiO_2_ thin films, undoped^38,39^ or indium-doped,^40^ on GaAs have demonstrated polycrystalline rutile (R–TiO_2_) thin films with limited interface control. However, these samples were not doped with REIs, and the studies lacked a systematic analysis of growth phase selectivity and interface control, both of which are critical for preserving the quantum coherence of optically activated REIs. Moreover, the growth temperature exceeded 500 °C, which is detrimental for QD functionality.

In this study, we demonstrate low-temperature heteroepitaxial growth of Er^3+^/TiO_2_ thin films on GaAs and GaSb substrates using PLD, with a focus on phase selectivity, interface control, and the optical activity of erbium ions. We introduce interface preparation steps that facilitate low-temperature (below 400 °C) growth of crystalline thin films with <300⁡pm roughness, compatible with low-loss nanophotonic structures for coherent spin-photon interactions. The crystalline phase of the thin films can be tuned between the anatase (A–TiO_2_) and rutile phases (R–TiO_2_) by controlling either the growth temperature or adapting the interface preparation steps. Minimal coincident interface area (MCIA) analysis explains the orientation-selective growth of anatase (001) on GaAs. Raman spectroscopy, cryogenic photoluminescence excitation (PLE), and electron microscopy confirm the crystal phase and the Er^3+^ optical activity. Together, these results establish an interface-conscious approach for monolithic integration of REIs with III–V semiconductors, laying the materials foundation for next-generation quantum photonic technologies.

## 2 Results and Discussion

### 2.1 Growth of Smooth TiO_2_ Thin Films

Er^3+^/TiO_2_ thin films were synthesized using PLD employing a KrF excimer laser (see Supporting Information, Section S1.1, for details). A rectangular aperture in a projection beamline defined a quasi-tophat beam profile, allowing a uniform fluence of 2.0 J·cm^–2^ over the illuminated area of the target. The resulting growth rate was approximately 0.17 Å per laser shot, as determined by postgrowth profilometry and validated by transmission electron microscopy (TEM). The substrates (5 × 5 mm^2^ chips) were mounted on a heated sample holder, and the chamber was evacuated to high vacuum (10^–6^ Torr). In the PLD chamber, all substrates were prepared using a thermal desorption procedure under a vacuum, with the desorption temperature chosen according to the initial surface termination. As shown in Figure 1, commercial epi-ready GaAs(100) substrates with unavoidable native-oxide termination were heated above 540 °C^41–44^ to desorb the oxide, while GaAs substrates terminated with a protective amorphous arsenic capping layer were heated to 350–365 °C^45^ to remove the As cap and expose a clean GaAs surface.

**Figure 1:**
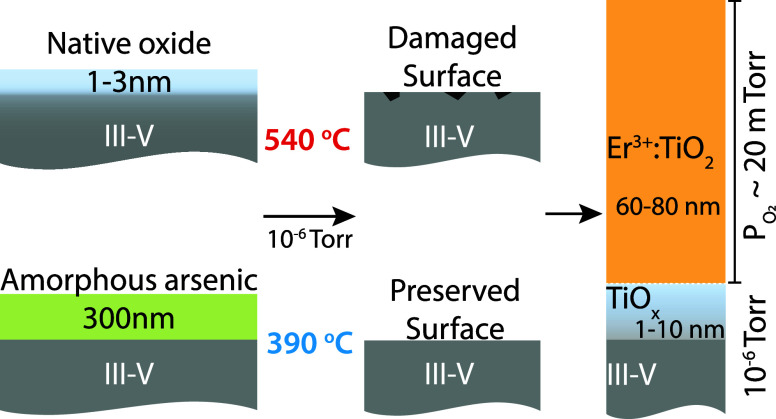
Schematic of the PLD process for synthesizing Er^3+^/TiO_2_ thin films on III–V substrates, highlighting surface preparation for arsenic-capped and uncapped wafers.

Molecular beam epitaxy was used to prepare native-oxide-free GaAs surfaces with well-defined reconstruction, which were subsequently protected by an amorphous arsenic cap prior to PLD growth. Arsenic-capped GaAs wafers were prepared by heating epi-ready GaAs(100) substrates to 620 °C under ultrahigh vacuum to remove native oxide. Oxide removal was confirmed using reflection high-energy electron diffraction (RHEED), after which a 200 nm GaAs epilayer was grown to reduce surface roughness and restore a high-quality crystalline surface. The substrate temperature was then lowered, and an amorphous As cap was deposited at room temperature to prevent reoxidation during subsequent exposure to ambient conditions.

Other substrates included in our study (GaSb(100), silicon(100)-on-insulator, and R–TiO_2_(110)) were prepared by high-temperature thermal treatment until well-defined RHEED patterns were observed in the PLD chamber. A thin TiO_*x*_ buffer layer is intentionally deposited under a high vacuum (10^–6^ Torr) at the onset of growth to suppress reoxidation of the freshly prepared III–V surface. The oxygen pressure was then raised to 20 mTorr for the remainder of the thin film growth. This increased pressure restores the stoichiometry of the TiO_2_ thin film by limiting the formation of oxygen vacancies. In addition, collisions between oxygen molecules and the plasma plume reduce the kinetic energy of Ti^4+^ species, minimizing ionic bombardment of the substrate and potential damage to the film surface. This two-stage oxygen pressure sequence promotes the synthesis of well-oxidized TiO_2_ thin films.^46^ Each film, 60–80 nm thick, was grown within ∼20 min, followed by a short (30 min) oxygen anneal during cooldown. Samples are labeled according to their substrate and general high or low growth temperature (HT or LT, respectively; see Supporting Information, Section S1.1, for full sample growth conditions).

The crystalline quality of the films was monitored in situ by reflection high-energy electron diffraction (RHEED) in two stages: after substrate surface preparation and following TiO_2_ film growth. For oxide-desorbed GaAs (Figure 2a,b), the diffraction patterns exhibit weak Kikuchi lines and pronounced spotty features, indicative of a roughened surface and island-like reconstruction reported earlier.^41,42^ In contrast, GaAs substrates prepared with an arsenic cap (Figure 2e,f) yielded streaky RHEED patterns with sharper Kikuchi lines, confirming the recovery of a smoother and ordered GaAs(100) surface compatible with epitaxial film growth even at low desorption temperatures (350 °C). Films grown on uncapped substrates displayed weaker features consistent with degraded interface quality (Figure 2c,d) in comparison to films grown on arsenic-capped GaAs (Figure 2g,h), whose postgrowth RHEED images of TiO_2_ exhibited vertical streaks characteristic of predominantly two-dimensional growth and good crystallinity. The presence of superimposed spots suggests contributions from step edges or islands resulting from buried crystal defects. Notably, the period of the RHEED features is significantly different between the two growth processes, indicating different crystal structures or phases of the TiO_2_ thin films.

**Figure 2:**
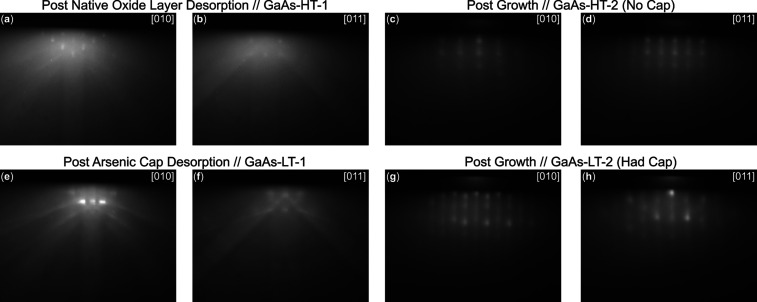
Images of the epi-ready GaAs substrate (sample GaAs–HT-1) after native oxide layer desorption are shown in (a,b) for the [010] and [011] directions, respectively. RHEED patterns for a separate sample (GaAs–HT-2) after ∼90 nm of TiO_2_ growth utilizing a similar epi-ready GaAs substrate is shown in (c,d) for the [010] and [011] directions, respectively. Images of the GaAs substrate (sample GaAs–LT-1) after desorption of the amorphous arsenic cap are shown in (e,f) for the [010] and [011] directions, respectively. RHEED patterns for a separate sample (GaAs–LT-2) after ∼60 nm of TiO_2_ growth also utilizing an amorphous arsenic-capped GaAs substrate is shown in (g,h) for the [010] and [011] directions, respectively.

Surface morphology was quantified by atomic force microscopy (AFM). TiO_2_ films grown after oxide desorption of uncapped GaAs substrates displayed irregular surfaces with some particle agglomeration, yielding root-mean-square (RMS) roughness values exceeding 2 nm (Figure 3a). In stark contrast, TiO_2_ films grown on capped GaAs at *T*_grow_ ≈ 390 °C consistently exhibited smooth surfaces with subnanometer roughness. The lowest surface RMS roughness measured over a 5 × 5 μm^2^ area was 116 pm from the AFM scan shown in Figure 3b (sample: GaAs–LT-3). The average RMS roughness estimated across several scans was ≈378pm on this sample.

**Figure 3:**
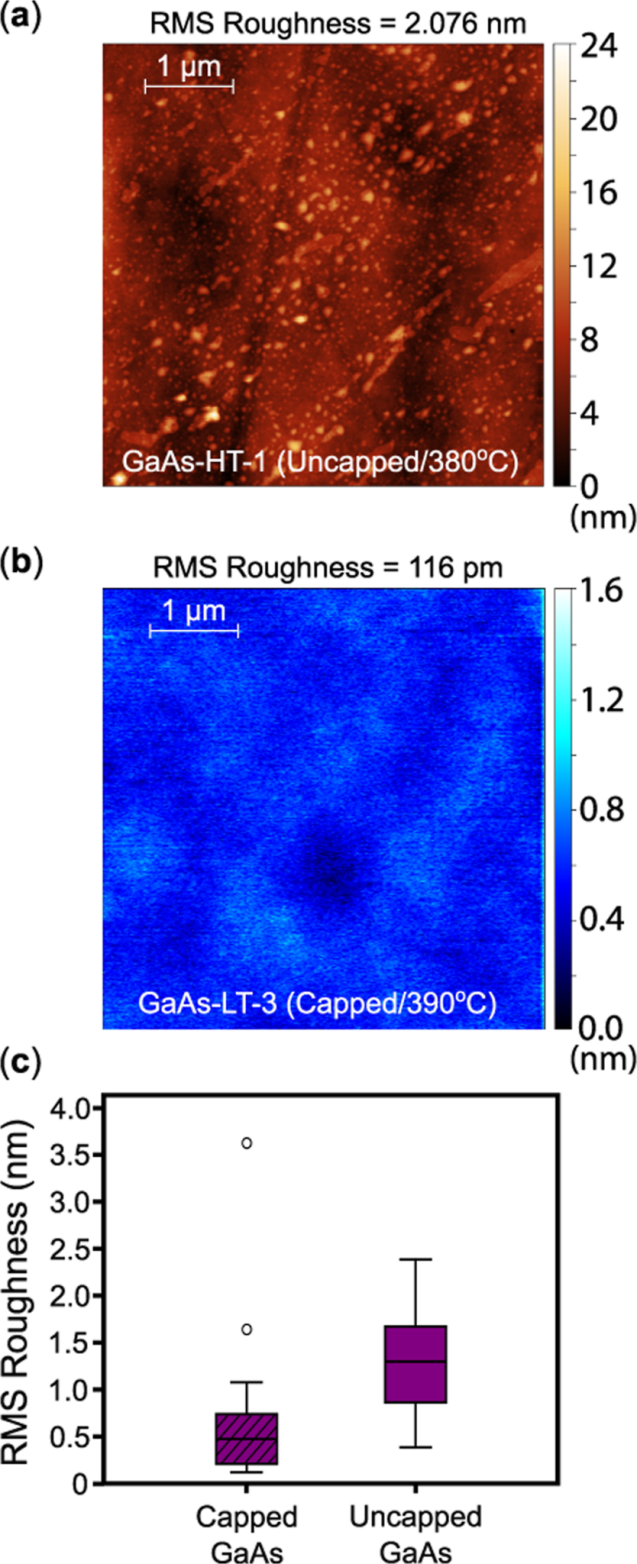
Example AFM scan after postprocessing for a (a) high-temperature (R–TiO_2_)-GaAs sample (GaAs–HT-1) synthesized after desorbing the native oxide layer present on the GaAs substrate and a (b) low-temperature (A–TiO_2_)-GaAs sample (GaAs–LT-3) utilizing a protective amorphous arsenic cap. (c) Box-and-whisker plot of mean RMS roughness values extracted across multiple scans for capped and uncapped GaAs substrates.

Statistical analysis of surface roughness measured over a 5 × 5 μm^2^ area for samples utilizing arsenic-capped GaAs and uncapped GaAs substrates is summarized in Figure 3c. Our study confirms that capped GaAs reproducibly yields the smoothest films, while uncapped GaAs substrates typically produce rougher surfaces. Full sample composition details are available in Supporting Information, Section S1.1. We emphasize that not all synthesized films possess a fully Er^3+^-doped layer after the initial undoped buffer layer, as shown in Figure 1. In these other cases, the remaining film is either undoped or “sandwich” doped, that is, only a small 2–10 nm section of the remaining film, positioned between undoped TiO_2_ layers, is doped.

At the optimal growth temperature of 390 °C on arsenic-capped GaAs, the majority of TiO_2_ films yielded subnanometer RMS roughness (200–600 pm). This reproducibility highlights the robustness of the capping strategy in producing smooth surfaces suitable for nanophotonic integration. We note, however, two outliers with RMS roughness above 1 nm on capped GaAs growth (see Supporting Information, Section S1.4, for sample details): GaAs–LT-4 (*T*_grow_ = 400 °C) and GaAs–LT-5 (*T*_grow_ = 350 °C). The roughness of the thin film on GaAs–LT-4 (grown at 400 °C) exhibited surface contamination likely introduced during transfer to the PLD chamber, and the unusually rough surface of GaAs–LT-5 is due to incomplete desorption of the arsenic cap at the reduced growth temperature (350 °C). Further information for additional samples synthesized with GaSb and silicon-on-insulator (SOI) substrates is included in Supporting Information, Section S2.1.

### 2.2 Optical Activation of Er^3+^ in TiO_2_ Thin Films

A key question is whether Er^3+^ ions remain optically active under direct TiO_2_/III–V integration and how this optical activity depends on the surface preparation and growth conditions. To address this, we combine Raman spectroscopy, which fingerprints the TiO_2_ crystal phase, with photoluminescence excitation (PLE) spectroscopy of the *Z*_1_ → *Y*_1_ transition, which is a sensitive probe of Er^3+^ optical activation. Raman spectra were collected at room temperature under 514 nm laser excitation in a commercial system (Renishaw inVia). PLE measurements were performed at 5.2 K using a tunable telecom-band laser and time-gated single-photon detection in a confocal microscopy setup. Photoluminescence collected from the sample was spectrally filtered with a 1400 nm long-pass filter, which selectively transmitted Er^3+^ emission from the ^4^I_13/2_ manifold following laser excitation from the crystal-field split *Z*_1_ state of the ^4^I_15/2_ manifold. This detection scheme suppresses scattered laser light, ambient background, and upconversion fluorescence, while time gating further rejects short-lived background emission and enhances the signal-to-noise ratio for resolving narrow Er^3+^ optical transitions. Complete experimental details of the PLE setup are provided in Supporting Information, Section S4.2.

Across the Er^3+^/TiO_2_ thin film samples, two distinct optical signatures emerged, correlating with the substrate preparation and growth conditions. Films synthesized at elevated substrate temperatures (≥450 °C) on oxide-desorbed (uncapped) III–V substrates crystallize in the rutile phase (R–TiO_2_), as confirmed by Raman spectra exhibiting the characteristic *E*_g_ (449 cm^–1^) and A_1g_ (614 cm^–1^) modes (Figure 4a). Additional spectral features seen around 1300 cm^–1^ is the visible fluorescence of Er^3+^ ions under 514 nm excitation. Specifically, the 4f-electrons are excited into the ^4^S_3/2_ or ^2^H_11/2_ manifolds, which relax via a combination of phonon-assisted and radiative processes to ^4^I_15/2_, producing visible fluorescence around 550 nm ( $∼1300{\;cm}^{-1}$ ). While this fluorescence validates the incorporation of Er^3+^ into the TiO_2_ lattice, visible emission is intrinsically strongly phonon-coupled and is therefore unsuitable for line width or coherence analysis. Consistent with the rutile phase identified from Raman spectra, cryogenic PLE measurements reveal a sharp crystal-field split *Z*_1_ → *Y*_1_ resonance of the ^4^I_13/2_ → ^4^I_15/2_ transition at 197.16 THz (1520.5 nm), characteristic of Er^3+^ ions substituting at Ti^4+^ sites in the R–TiO_2_ lattice^47^ (Figure 4b). These observations establish that Er^3+^ remains optically active at telecom wavelengths in rutile TiO_2_ films grown directly on oxide-desorbed III–V substrates.

**Figure 4:**
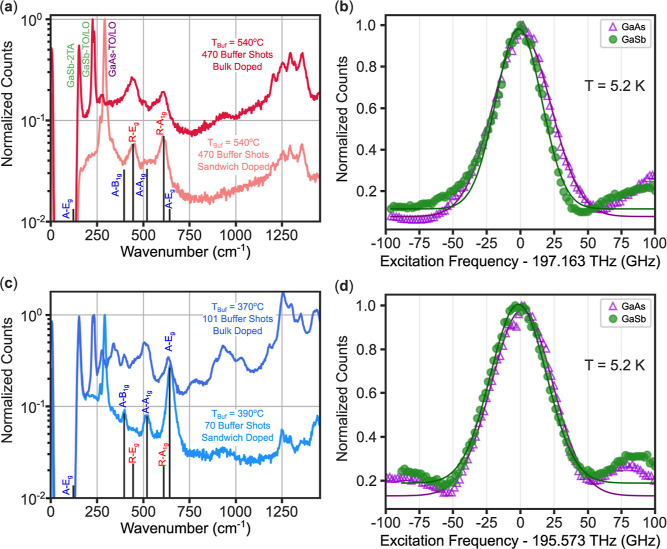
(a) Raman spectroscopy results for two high temperature (R–TiO_2_)-III–V samples. The dark red trace is sample GaSb–HT-1 (bulk-doped; GaSb substrate), and the light red trace is sample GaAs–HT-3 (sandwich-doped; GaAs substrate). Sample buffer shots and buffer growth temperatures are included within the figure. (b) *Z*_1_ → *Y*_1_ Er^3+^ PLE results for samples GaAs–HT-4 (purple) and GaSb–HT-1 (green). (c) Raman spectroscopy results for two low temperature (A–TiO_2_)–III–V samples. The dark blue trace is sample GaSb–LT-1 (bulk-doped; GaSb substrate), and the light blue trace is sample ST2417 (sandwich-doped; GaAs substrate). Sample buffer shots and buffer growth temperatures are included within the figure. (d) *Z*_1_ → *Y*_1_ Er^3+^ PLE results for samples GaAs–LT-1 (purple) and GaSb–LT-1 (green).

In contrast, films grown at lower substrate temperatures (370–390 °C) on arsenic-capped GaAs stabilize in the anatase phase (A–TiO_2_). Raman spectra exhibit the characteristic B_1g_, A_1g_, and *E*_g_ modes of anatase, including a pronounced low-frequency *E*_g_ peak near 144 cm^–1^ indicative of good crystallinity (Figure 4c). Corresponding cryogenic PLE spectra reveal a systematic shift of the Er^3+^ *Z*_1_ → *Y*_1_ transition to 195.57 THz (1532.9 nm), consistent with Er^3+^ substitution at Ti^4+^ sites in the anatase lattice^48^ (Figure 4d). The correlated frequency shift observed in both Raman and PLE measurements directly links the optical response of Er^3+^ to the TiO_2_ crystal phase selected by interface preparation and growth conditions.

Another pronounced optical distinction between capped and uncapped growth is observed in the excited-state lifetimes (see Supporting Information, Section S4.3). Er^3+^ doped into R–TiO_2_ films grown at elevated temperature on uncapped III–V substrates exhibit optical lifetimes of 4.7–5.3 ms, which drops to 1.3–1.7 ms in A–TiO_2_ films stabilized under low-temperature growth. This approximately 3-fold reduction highlights the strong sensitivity of Er^3+^ decay dynamics to the crystal phase selected by the growth regime and surface preparation. These differences in lifetime are consistent with reported values of A–TiO_2_ ^22,23^ and R–TiO_2_ thin films.^35^ In thin-film geometries, such phase- and growth-dependent lifetime changes arise from modifications to both radiative decay rates and nonradiative pathways. While the crystal field and the local density of optical states near interfaces influence the radiative decay rate,^14,17,19^ nonradiative decay is enhanced by point defects and oxygen-vacancy-related quenching.

Interestingly, each growth regime also exhibited more subtle substrate-dependent variations. Both rutile and anatase films grown on GaSb consistently exhibit narrower Er^3+^ inhomogeneous line widths (Figure 4b,d) and shorter excited-state lifetimes than those grown on GaAs. For example, PLE scans for rutile films shown in Figure 4b reveal that the line width narrows from 50.9(7) GHz on GaAs to 40(1) GHz on GaSb. However, the lifetime decreases from ∼5.3(3)⁡ms on GaAs to ∼4.7(2)⁡ms on GaSb. Such decoupling of line width and lifetime has been observed in other REI-doped oxides, where ensemble inhomogeneity is dominated by static disorder (strain fields, compositional fluctuations), while the homogeneous line width and lifetime are set by dynamic decoherence and nonradiative processes (spin flips, spectral diffusion, or defect-assisted relaxation).^49,50^ Similarly, photon-echo studies demonstrated that magnetic-noise-induced spectral diffusion can broaden homogeneous line widths without altering ensemble disorder.^10,51^ Therefore, we conclude that the observed small variations indicate interface-specific nonradiative decay pathways that depend on the local chemical and structural environment experienced by Er^3+^ ions near the oxide/III–V interface. Likely contributors include gallium diffusion into the oxide lattice,^52,53^ differences in the chemistries of As- and Sb-terminated interfaces,^54^ oxygen-vacancy-mediated quenching processes,^55,56^ and local strain relaxation that can modify the crystal-field splitting with interfacial distance.

We emphasize that the inhomogeneous line widths measured here reflect ensemble-level disorder and do not directly constrain the optical coherence time (*T*_2_) of Er^3+^ ions, which is governed by spectral diffusion, phonon coupling, and local electromagnetic noise. Direct measurements of homogeneous line widths and *T*_2_ times will require future photon-echo experiments. Recent first-principles studies have uncovered the role of interfacial gallium diffusion, oxygen-vacancy dynamics, and strain-induced crystal-field modulation in shaping the optical properties of Er^3+^/TiO_2_/III–V heterostructures.^57^ Nevertheless, the observation of narrow optical transitions at high (>1000 ppm) doping concentrations demonstrates that Er^3+^ ions remain optically active under both capped and uncapped growth conditions. In particular, arsenic-capped, low-temperature growth enables smooth A–TiO_2_ films that preserve the Er^3+^ optical activation in monolithic TiO_2_/III–V platforms suitable for low-loss nanophotonics.

Having established the optical activation of Er^3+^ across growth regimes, we now turn to the material-growth factors governing the phase selectivity in TiO_2_ thin films. PLE and Raman spectra on representative samples in Figure 4 show that a combination of controlled surface preparation and growth temperature within a two-stage oxygen pressure process enables the stabilization of anatase TiO_2_ on GaAs, extending earlier PLD studies that reported exclusively rutile-phase films.^38,39^ Moreover, the same growth process also supports the synthesize of both rutile- and anatase-phase TiO_2_ on GaSb, an emerging platform for telecom-band single-photon sources.^58^ Building on these spectroscopic benchmarks, TiO_2_ phase selectivity is examined across a broader growth parameter space comprising more than 20 samples grown on arsenic-capped GaAs, uncapped GaAs, and GaSb substrates (see Supporting Information, Section S1.1, for sample details). We observe that the phase selectivity of TiO_2_ is not determined by the growth temperature alone but may also be influenced by buffer-layer thickness in certain cases, as suggested by the phase diagram (Figure 5a). Notably, two samples grown on capped-GaAs substrates at 390 °C, but with different buffer thicknesses, yielded distinct phases: a thinner buffer (1.2 nm) stabilized the anatase phase, while a thicker buffer (8.5 nm) drove rutile formation. The Raman spectra (Figure 5b) clearly illustrate this contrast, with the thick buffer sample showing a complete suppression of the 144 cm^–1^ *E*_g_ mode. This anomalous growth suggests that strain relaxation and the accumulation of oxygen vacancies beyond a critical buffer thickness can tip the balance toward rutile even at reduced growth temperatures. While this single observation is insufficient to establish a general trend, it suggests that buffer engineering may play a role in controlling phase stability and merits future investigation. To provide broader insight into phase selectivity and film quality, we next examine the microstructural and interfacial characteristics of representative films.

**Figure 5:**
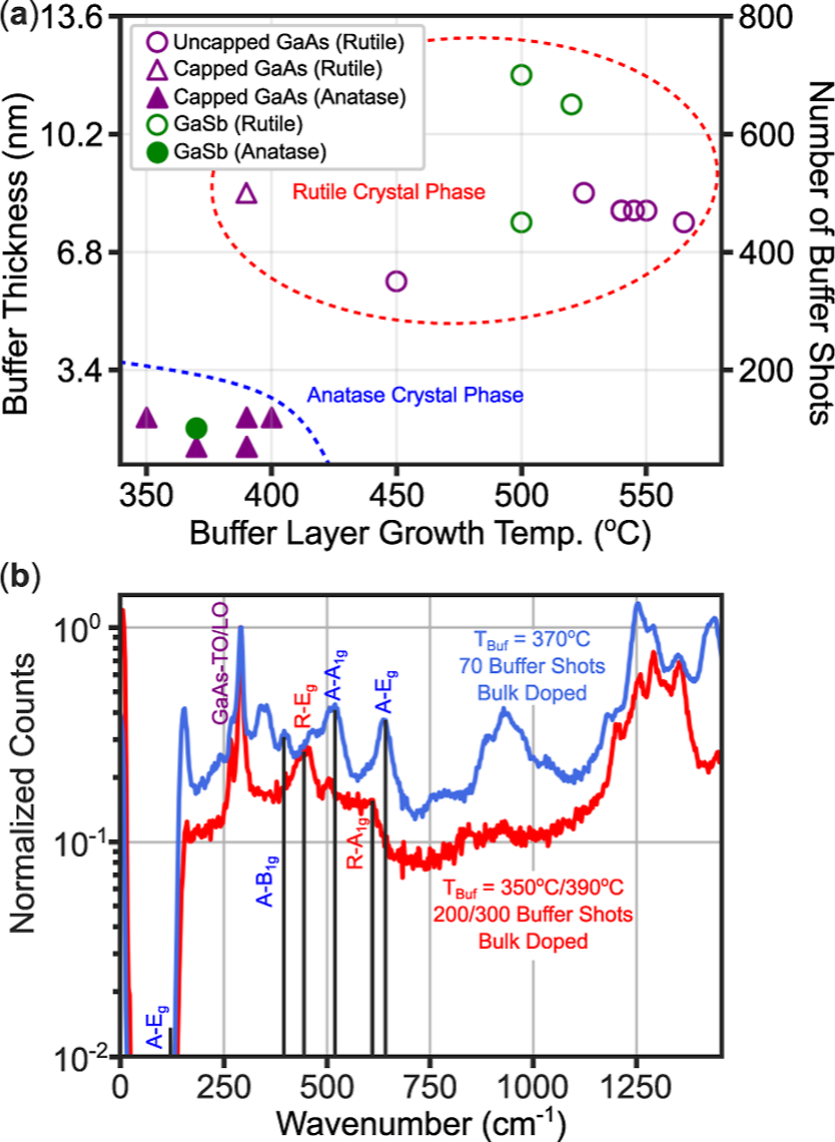
(a) Phase diagram for all synthesized TiO_2_ thin films on GaAs and GaSb substrates. The buffer thickness is calculated by multiplying the number of laser shots fired before purging oxygen into the chamber (“number of buffer shots”) by the estimated growth rate of 0.17 Å per laser shot. (b) Raman spectra for the open purple triangle R–TiO_2_ film on GaAs substrate (red trace; sample GaAs–LT-6) data point in (a) along with a bulk-doped A–TiO_2_ film on GaAs substrate (blue trace; sample GaAs–LT-1) for comparison. Relevant phonon modes with drop lines to guide the eye are labeled for GaAs, A–TiO_2_ (blue “A” prefix), and R–TiO_2_ (red “R” prefix).

### 2.3 Crystallographic Phase and Microstructure Analysis

The crystallographic orientation and microstructure of the TiO_2_ films were investigated by using minimum coincident interface area (MCIA) modeling in conjunction with θ–2θ X-ray diffraction (XRD) measurements. MCIA provides a geometric metric for predicting orientation-selective epitaxy by quantifying the smallest lattice-commensurate overlap between a film and substrate,^59,60^ while XRD directly proves the resulting out-of-plane order, grain size, and strain. Together, these methods establish the connection between the interface energetics and the observed structural phase. Note that because MCIA assumes atomically sharp, defect-free interfaces, which are challenging to realize in PLD, it serves as a predictive metric rather than a guarantee. In practice, maintaining a high-quality interface, such as through arsenic capping, is essential for approaching the geometric minimum.

The MCIA maps in Figure 6 reveal clear orientation-dependent trends consistent with experimentally observed phase selectivity. For GaAs, anatase (001) exhibits the smallest MCIA value (64 Å^2^), nearly an order of magnitude lower than that of rutile (110). Such a small MCIA value suggests that even moderate interfacial disorder or step-edge roughness can still support anatase-phase epitaxy at lower growth temperatures, consistent with surface-energy minimization predicted by MCIA. At higher temperatures (>450 °C), enhanced adatom mobility and oxygen incorporation promote atomic rearrangement toward minimizing bulk energy, favoring the thermodynamically denser rutile phase. However, rutile (110) and (210) are nearly degenerate in MCIA, resulting in mixed-texture films observed at elevated growth temperatures.^38,39^ For GaSb, both anatase (001) and rutile (001) orientations yield comparably small MCIA values $\left(\approx 175{\;Å}^{2}\right)$ , which implies that the energy balance between strain and chemical bonding, rather than pure lattice matching, determines which phase forms. By comparison, the MCIA values of anatase and rutile phases on Si(100) are comparable (300 Å^2^), consistent with the polycrystalline TiO_2_ films typically reported in the literature.^22,23^ These geometric trends provide a predictive framework linking the interface geometry to the phase selectivity observed in Figure 5, motivating the experimental validation of the out-of-plane crystal orientation using XRD. Establishing a full epitaxial relationship would require complementary in-plane XRD measurements (e.g., pole figures). However, such measurements are beyond the scope of this work, which focuses on validating the out-of-plane orientations predicted by the geometric model.

**Figure 6:**
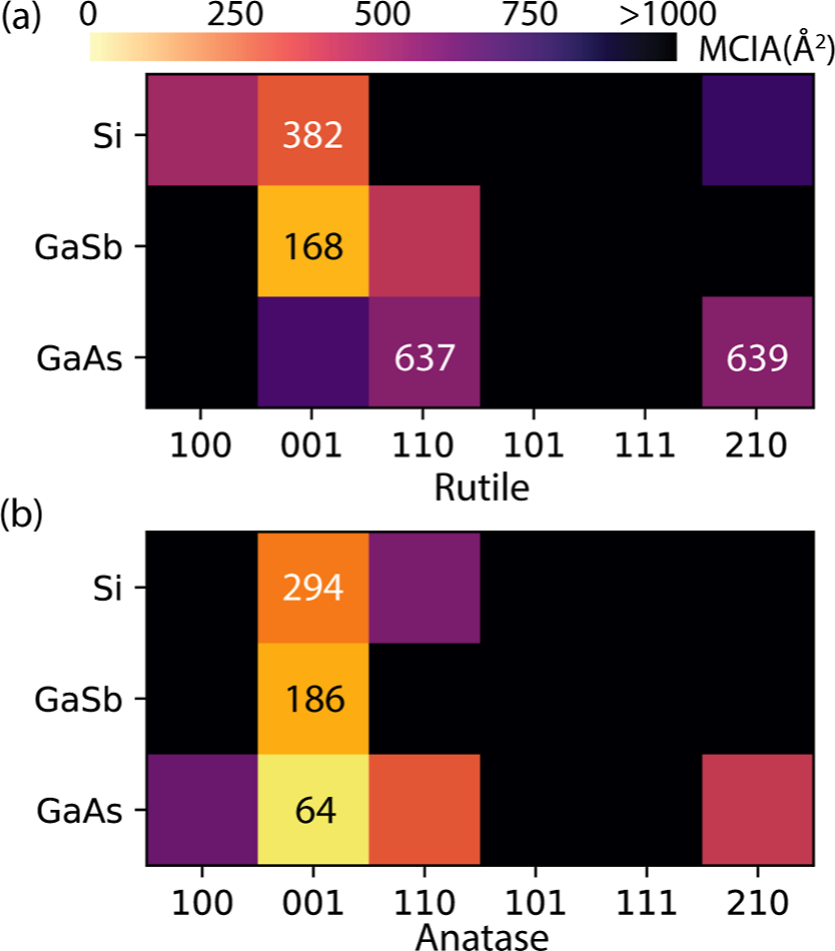
Calculated minimal coincident interface area (MCIA) for (100)-oriented Si, GaSb, and GaAs substrates with different crystal orientations, labeled by the corresponding Miller index, of (a) rutile and (b) anatase phase TiO_2_. The smallest MCIA values for a given substrate–film pair are marked in the plot.

A representative θ–2θ XRD scan of a TiO_2_ thin film synthesized on oxide-desorbed GaAs at high growth temperatures (565 °C) is shown in Figure 7a. The dominant reflection peak at 2θ = 27.4° matches the rutile (110) plane, in agreement with the MCIA predictions. The corresponding (220) harmonic and a weak (210) reflection are also observed, with the relative intensities suggesting a partial preferred orientation rather than a fully random polycrystalline structure. Using the Scherrer and Wilson equations upon fitting the (110) peak to a Voigt line shape (Figure 7b), the extracted grain size (τ) and microstrain (ϵ) were determined to be 22 ± 1 nm and 0.68 ± 0.04%, respectively. Given the estimated film thickness of ∼90 nm for high-temperature growths, the ∼22⁡nm grain size confirms that the R–TiO_2_ films are polycrystalline, consistent with previous reports.^38,39^ The nonzero ϵ indicates that even after grain breakup a residual tensile component remains within the film. The overall trend of small, tensile-strained crystallites is consistent across all high-temperature samples, with τ and ϵ ranging from 14–32 nm and 0.47–0.96%, respectively (see Supporting Information, Section S2.5, for details). These microstructural characteristics (polycrystalline morphology, partial preferred orientation, and residual tensile strain) are consistent with the high and nearly degenerate MCIA values predicted for (110)/(210) R–TiO_2_ orientations on GaAs.

**Figure 7:**
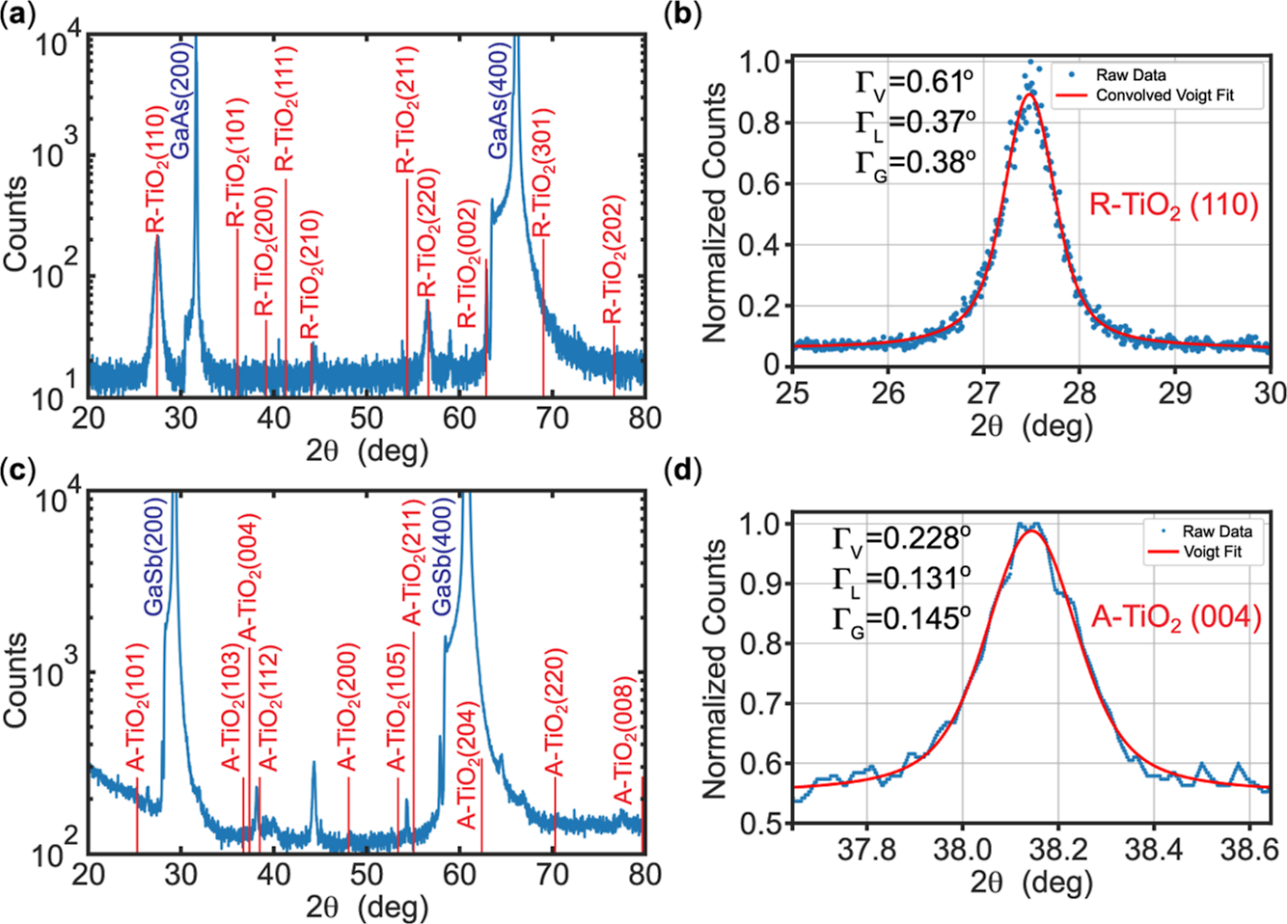
(a,b) XRD spectra for sample GaAs–HT-2. (a) Wide θ–2θ scan of the sample. Vertical red lines indicate the expected R–TiO_2_ peak positions of a perfect crystal using a CuK_α1_ X-ray source. (b) Zoom-in of the R–TiO_2_ (110) XRD peak and its resulting convolved Voigt fit. (c,d) XRD results for the (A–TiO_2_)–GaSb sample (GaSb–LT-1). (c) θ–2θ XRD spectra. Vertical red lines indicate the expected A–TiO_2_ peak positions of a perfect crystal using a CuK_α1_ X-ray source. (d) Zoom-in of the A–TiO_2_ (004) XRD peak and its resulting Voigt fit.

At lower growth temperatures (<390 °C), the XRD signal from TiO_2_ grown on arsenic-capped GaAs was too weak to unambiguously resolve the diffraction peaks under standard θ–2θ geometry, likely due to the film’s limited thickness (≈60⁡nm) and low scattering volume.^61^ Nevertheless, the smooth AFM morphology and distinct Raman signatures (Figure 5) indicate crystalline anatase formation. Grazing-incidence XRD measurements further revealed a weak but reproducible A–TiO_2_ (101) reflection (Supporting Information, Figure S2), providing additional confirmation of the anatase phase despite the limited film thickness. A measurable XRD signal was also obtained for an analogous film grown on GaSb in the same temperature range (Figure 7c). A narrow A-TiO_2_(004) reflection is evident (Figure 7d), confirming anatase-phase stabilization under diffusion-limited growth conditions. Voigt-profile fitting yields a grain size of 64(2) nm, comparable to the film thickness, and a microstrain of ϵ = 0.183(6)%. The fitted Voigt line width is nearly a third of the high-temperature rutile film on GaAs (Figure 7b), consistent with a substantial reduction in microstrain broadening and overall improvement in crystalline quality. The extracted A(004) peak center of 38.144(1)° corresponds to a lattice parameter *c* = 9.43(1) Å, indicating a minor compressive strain consistent with the interfacial registry predicted by MCIA. Overall, the transition from polycrystalline, tensile-strained rutile to smooth, low-strain anatase with decreasing growth temperature aligns with the MCIA predictions and thermodynamic trends discussed earlier.

### 2.4 Interface Chemistry and Defects

Building on the structural and phase evolution described above, we next examined the atomic-scale interface chemistry and defect structure that govern TiO_2_-(III–V) heteroepitaxy. The transition from tensile-strained rutile to relaxed anatase, together with the sensitivity to substrate termination, indicates that interfacial bonding and stoichiometry critically determine both phase stability and optical performance. To elucidate these effects, we performed cross-sectional transmission electron microscopy (TEM) and electron energy-loss spectroscopy (EELS) on a sandwich-doped oxide-desorbed GaAs sample with an R–TiO_2_ film (sample GaAs–HT-5; see Supporting Information, Section S1.1, for full composition details). This sample contained a 10 nm oxygen-deficient TiO_*x*_ buffer grown at 545 °C, followed by an 80 nm TiO_2_ layer deposited at 20 mTorr oxygen pressure. A central 10 nm region of the film was selectively doped with erbium using a 3000 ppm Er^3+^/TiO_2_ target, intended to trace possible Er^3+^ migration along the growth detection. Although this sample represents a single high-temperature growth, it serves as a detailed case study for understanding interface reactions and defect formation in the rutile regime.

Scanning transmission electron microscopy (STEM) imaging was utilized to study the film morphology. Both high-angle and low-angle annular dark field (HAADF and LAADF, respectively) STEM were employed (Figure 8a,b). HAADF contrast is well-established to originate primarily from atomic mass, with heavier atoms appearing brighter, whereas LAADF is largely influenced by diffraction contrast.^62^ This manifests as the HAADF image (Figure 8a) providing directly interpretable contrast information (relating primarily to atomic number with minimal diffraction contributions), whereas LAADF imaging (Figure 8b) highlights the presence of intercolumn crystal rotation. The GaAs substrate exhibits dark/bright pits and an uneven surface topography consistent with the oxide-desorption-induced damage. High-resolution TEM (HRTEM) and STEM images of these nanoscale depressions (Figures S6 and S7 of Supporting Information) indicate gallium-deficient voids, where disrupted surface reconstruction locally alters the nucleation density and promotes nonuniform columnar alignment of the TiO_2_ film. The oxygen-deficient buffer can be distinguished from the overlying TiO_2_ layer by discontinuity in the columnar texture. The high-resolution inset from Figure 8b (red box) shown in Figure 8c reveals the twisting and reorientation of atomic planes between neighboring regions, indicating the presence of misoriented crystal domains throughout the film. The Moiré fringes observed throughout the wide-view HRTEM image in Figure 8d arise from this crystal misorientation when viewed in the projection. These fringes extend through the entire film thickness, reflecting the complex rotational variants occurring on the few-nanometer scale. Similar patterns were observed at multiple regions across the cross-section, confirming that crystal misorientation is a pervasive microstructural feature. The frequent observation of both in-plane and out-of-plane rotations within the <100 nm thick lamella suggests the presence of small grains throughout the film (additional examples in Supporting Information, Section S2.8).

**Figure 8:**
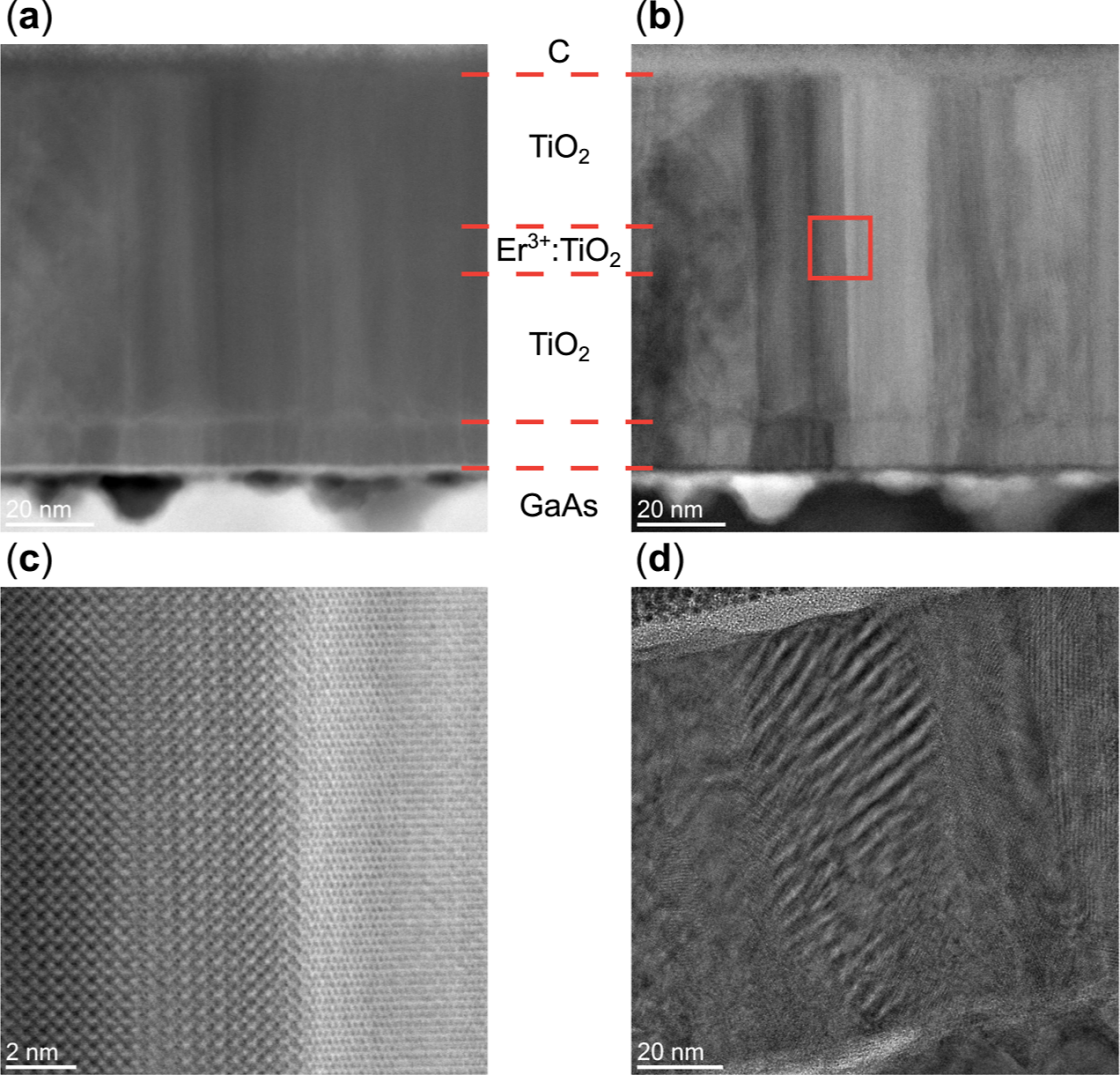
TEM images of the (Er^3+^/R–TiO_2_)–GaAs sample. (a) HAADF–STEM image showing the columnar structure of the TiO_2_ film. (b) Complementary LAADF–STEM image further highlighting the film’s composition as a function of growth position with dashed lines. (c) The inset from (b) shows the polycrystalline nature of the film and the atomic-level transition between grains. (d) HRTEM image of the TiO_2_ thin film. Moiré fringes where the crystal domains twist and overlap are clearly visible and extend over several tens of nanometers in the growth direction.

Atomic-resolution STEM imaging of the interfacial regions (Figure 9) provides insight into the structural origin of the observed columnar morphology and Moiré textures. An approximately 5 nm-wide interfacial region within the GaAs substrate exhibits pronounced contrast variation (Figure 9a), indicative of localized substrate damage induced during high-temperature oxide desorption. This lateral nonuniformity of the damaged GaAs surface disrupts uniform nucleation and grain coalescence during subsequent TiO_2_ growth, leading to the formation of small, misoriented columnar grains. Despite this, locally well-ordered crystalline domains are observed above less damaged substrate regions, as shown in the inset. The measured 3.25 Å lattice spacing of these well-ordered rutile (110) planes matches bulk R–TiO_2_ values, suggesting that strain relaxation occurs rapidly during the early stages of growth. However, these locally ordered domains coexist with widespread rotational disorder and misoriented grains, manifested as Moiré patterns in Figure 8c,d, which limits the development of long-range crystalline coherence. Taken together, these observations indicate that achieving large-area epitaxial TiO_2_ on GaAs requires both atomically flat, damage-free substrates and precise control of growth kinetics, parameters that remain challenging to decouple in PLD.

**Figure 9:**
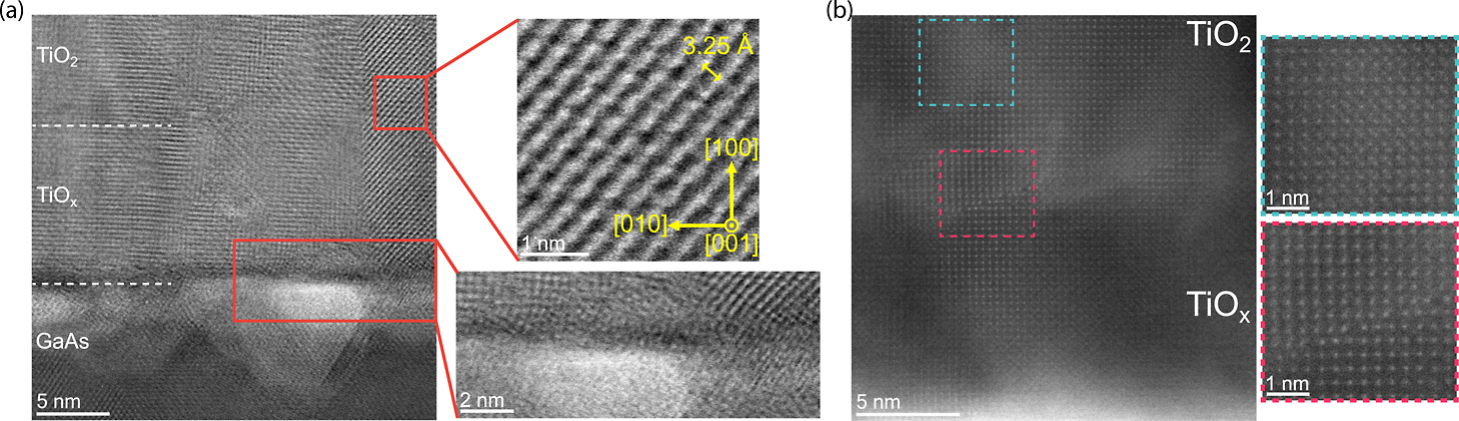
(a) Annular bright-field (ABF) image of the TiO_*x*_/TiO_2_/GaAs interface. Crystals grown at the undamaged GaAs substrate sites promote (110) R–TiO_2_ growth as shown in the top inset. The bottom inset shows a zoom-in of the damaged interface, and more specifically, a characteristic (gallium-deficient) pit (bright region) within the GaAs substrate and the beginning few monolayers of the TiO_*x*_ buffer layer. (b) HAADF–STEM image showing the atomic-level resolution of the TiO_*x*_/TiO_2_ interface. The blue inset shows an additional plane of atoms resulting from the rotation/twisting of the local crystal domain. The red inset shows the immediate surroundings of multiple vacancy centers, which cause lattice dislocations.

Atomic-resolution HAADF–STEM imaging of the narrow (1–2 nm) TiO_*x*_/TiO_2_ transition zone in Figure 9b reveals two characteristic features, indicated by the dashed boxes. The blue inset reveals multiple crystal planes that are slightly misoriented from the main lattice, consistent with the local rotation of crystal domains that produces Moiré fringes. The red inset highlights multiple lattice dislocations and accompanying vacancies, with a few atomic columns displaying enhanced contrast and irregular spacing, potentially corresponding to isolated gallium atoms incorporated during interdiffusion. Additional high-resolution STEM images of the TiO_*x*_/TiO_2_ interface and upper film regions, together with detailed scans of interfacial pits, are provided in the Supporting Information, Section S2.8. Although Er^3+^ ions could not be directly resolved within the 10 nm-doped region, this is expected, given their low concentration. Future STEM measurements on films with higher doping concentrations and enhanced signal-to-noise ratios may enable quantitative analysis of Er–O bond lengths and local substitution environments, including the influence of nearby oxygen vacancies.

Additionally, we performed cross-sectional TEM imaging of an A–TiO_2_ thin film sample (GaAs–LT-7) to directly compare growth on arsenic-capped and uncapped substrates. Figure 10 presents a HAADF–STEM image spanning the entire thickness of the TiO_2_ film, highlighting the compositionally distinct layers in the GaAs/TiO_*x*_/TiO_2_ stack. The structure consists of a 1.4 nm TiO_*x*_ buffer layer grown under vacuum and an approximately 60 nm TiO_2_ layer deposited at an oxygen pressure of 20 mTorr. A central 2 nm region of the film was selectively doped with erbium using a 3000 ppm of Er^3+^/TiO_2_ target. Due to the low Er^3+^ concentration and the thinness of the doped region, this layer is not directly resolved in the HAADF–STEM images.

**Figure 10:**
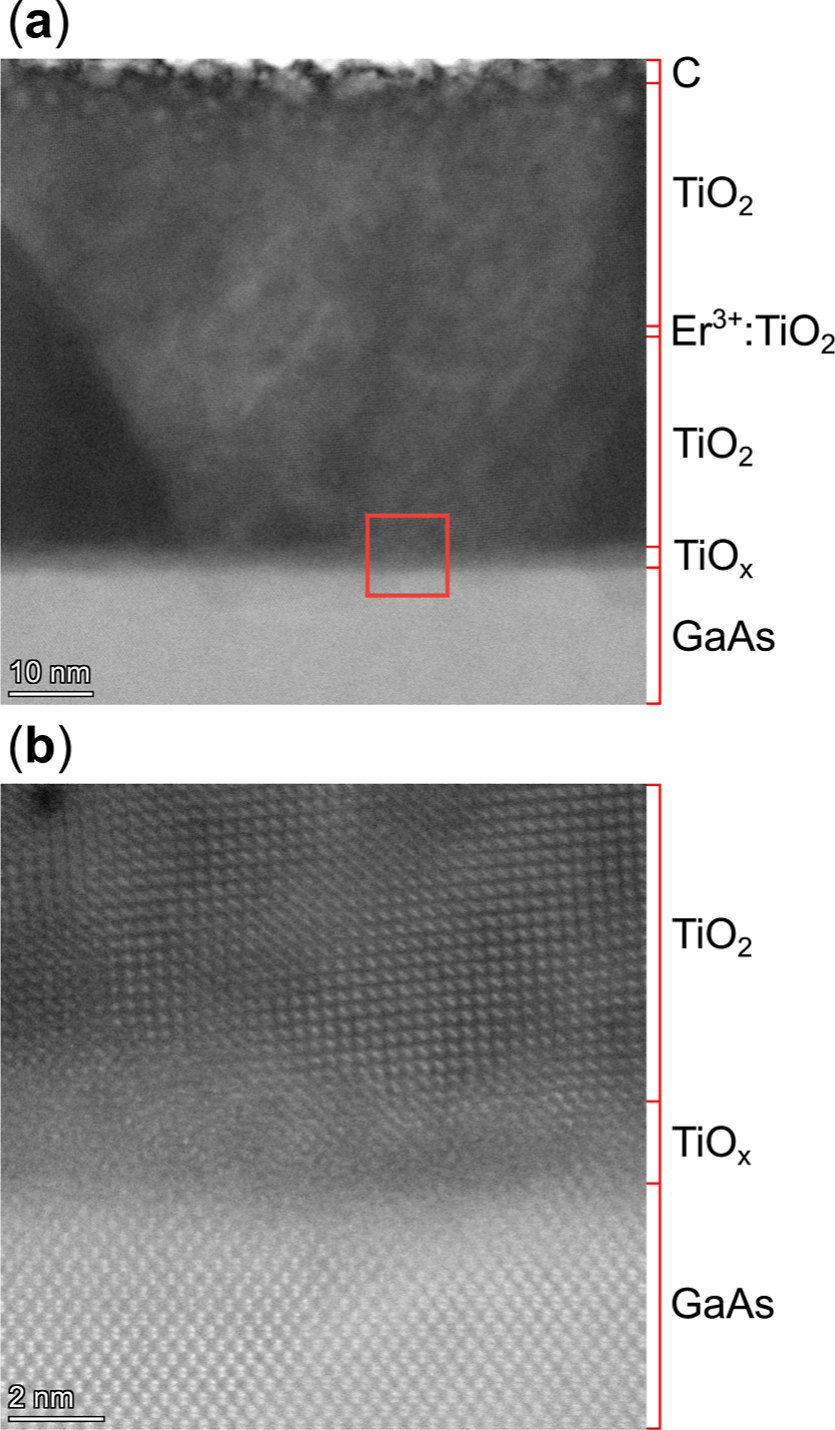
Cross-sectional HAADF–STEM analysis of an A–TiO_2_ thin film grown on arsenic-capped GaAs substrate (GaAs–LT-7). (a) Wide-field image spanning the full film thickness showing the compositional structure of the GaAs/TiO_*x*_/TiO_2_ layer stack. (b) Magnified atomic-scale resolution view of the TiO_*x*_/TiO_2_/GaAs interface (inset marked by red box in (a)), showing a smooth interface and an amorphized TiO_*x*_ buffer layer.

The wide-field HAADF–STEM image shown in Figure 10a reveals a markedly different microstructure compared with R–TiO_2_ films grown on uncapped GaAs (Figure 8a). In particular, the film lacks the pronounced columnar morphology observed on uncapped substrates, although localized in-plane twisting of crystal domains persists (Supporting Information, Figures S9 and S10). Crucially, the GaAs/TiO_*x*_/TiO_2_ interface is substantially smoother, with large regions free of the pits and deep nanoscale gallium-deficient features observed in uncapped substrates subjected to high-temperature oxide desorption (cf. Figures 8a,b and 9a). This improvement in interfacial morphology directly reflects the effectiveness of the arsenic-capping strategy, which preserves GaAs surface reconstruction and suppresses oxidation-induced damage prior to thin film deposition.

Figure 10b shows an atomic-scale resolution image of the GaAs/TiO_*x*_/TiO_2_ interface. Well-resolved atomic columns are observed in both the GaAs substrate and the overlying TiO_2_ layer, confirming good local crystalline order across the heterointerface. In contrast, the intervening TiO_*x*_ buffer layer appears amorphous, likely due to oxygen-deficient growth and subsequent chemical equilibration at the III–V interface. During growth on arsenic-capped substrates, the thickness of the buffer layer was intentionally limited to 1–1.5 nm, close to the minimum thickness at which TiO_2_ diffraction features first appear in RHEED. Although this approach enables an early transition to near-stoichiometric TiO_2_ growth while preserving the GaAs surface, the ultrathin interfacial layer is likely to undergo redox-driven restructuring and vacancy formation. This restructuring occurs during the deposition pause as the oxygen pressure increases to 20 mTorr (≈30⁡s) . The resulting amorphized TiO_*x*_ buffer layer disrupts the long-range epitaxial registry while still supporting the nucleation of locally well-ordered anatase TiO_2_ domains, consistent with MCIA predictions favoring A-TiO_2_(001) on GaAs. Further improvements may be achievable through careful control of plasma plume kinetics and slower oxygen-pressure ramps.^46^

Notably, despite the presence of an amorphous TiO_*x*_ transition layer, the GaAs substrate retains well-resolved atomic columns at the interface, indicating that the arsenic-capping strategy effectively preserves the underlying III–V lattice during oxide integration. This preserved interfacial integrity correlates with the smoother surfaces observed in AFM measurements with RMS roughness values below 300 pm. Together, these multiscale structural characterizations highlight the effectiveness of arsenic-capped substrates in enabling smooth, locally crystalline TiO_2_ films on GaAs.

To quantify the compositional gradients observed in STEM imaging, we performed EELS mapping of the R–TiO_2_ film grown on uncapped GaAs, as analyzed in Figures 8 and 9. The elemental distributions of Ga, As, Ti, and O (Figure 11a) delineate a sharp transition between the GaAs substrate and the TiO_2_ film, while the Er^3+^ signal remained below the detection threshold due to its low concentration. Depth-integrated line profiles, normalized to the maximum signal for each element (Figure 11b), reveal that Ti and O intensities increase abruptly at the interface, whereas Ga exhibits pronounced depletion on the substrate side, accompanied by correlated diffusion into the oxide film. A localized accumulation of Ga occurs near the TiO_*x*_/TiO_2_ boundary, corresponding to the region where growth was briefly halted during the oxygen-pressure ramp. This Ga migration behavior suggests that cation interdiffusion competes with oxygen incorporation during the transition from oxygen-deficient to stoichiometric growth. This process produces a thin compositionally graded zone enriched with Ga defects, which likely seeds the nonuniform columnar nucleation observed in STEM. Fitting the depth-resolved Ga profiles yields characteristic diffusion lengths of 4 nm and diffusion coefficients on the order of 10^–17^ cm^2^/s^–1^ (see Figure S8 of Supporting Information for details), consistent with low-activation-energy Ga diffusion in other oxide materials.^52,63,64^

**Figure 11:**
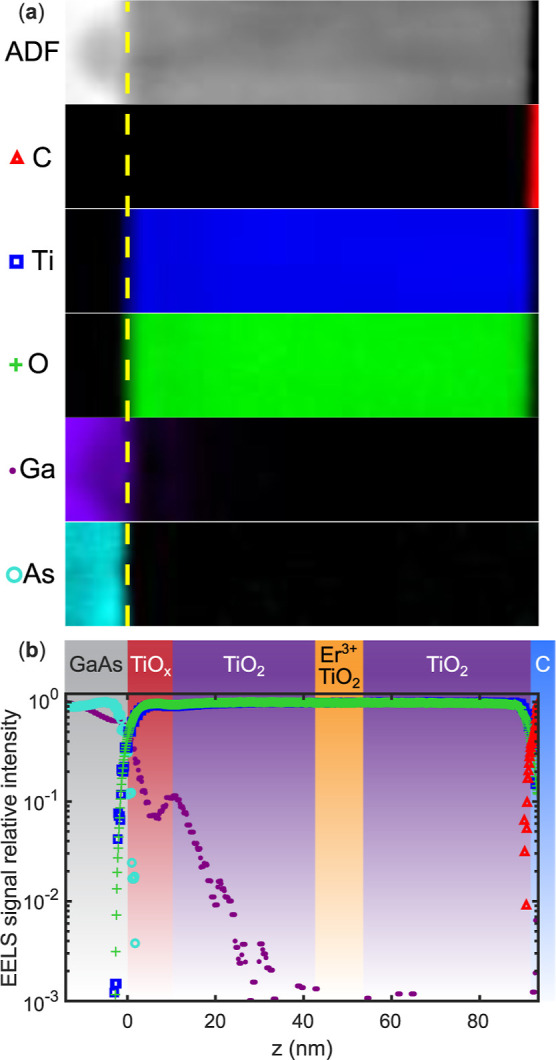
(a) Relative EELS signal intensity of each atom composing the TiO_2_/GaAs interface as a function of position from the GaAs substrate. (b) Integrated EELS signal of each element composing the TiO_2_/GaAs interface. Each elemental signal displayed is normalized to its column of pixels with the most integrated counts within each respective EELS map in (a). The carbon signal atop the TiO_2_ originates from the protective layer used during ion beam preparation of the TEM lamella.

Although the oxygen EELS signal varies with depth, the quality of the lamella in this region prevents the unambiguous separation of oxygen- and titanium-vacancy contributions near the interface. Beyond this region, a slight reduction in the oxygen signal relative to titanium indicates a finite population of residual oxygen vacancies that persist even after deposition and cooldown in the 20 mTorr oxygen pressure (see Figure S9 for details). To qualitatively assess their role, we modeled vacancy incorporation, diffusion, and annihilation during the oxygen-pressure ramp using a simple rate-equation framework (see Section S3.3 of Supporting Information).^55^ The simulations show that once the oxygen-deficient buffer exceeds a critical thickness, the average oxygen vacancy concentration saturates, limiting reoxidation, even after extended oxygen exposure. This residual vacancy population provides a plausible pathway for strain relaxation and polycrystalline rutile nucleation at lower growth temperatures, consistent with the phase diagram and microstructural observations above. Conceptually, the model supports a picture in which oxygen-vacancy accumulation couples interfacial chemistry to strain relief and phase stability in TiO_2_-(III–V) heterostructures.

### 2.5 Linking Microstructure to Optical Activation

The microstructural and interfacial trends described above provide a physical context for the optical activity of Er^3+^ discussed in Section 2.2. Residual strain and oxygen vacancies can each contribute to the inhomogeneous line widths by locally perturbing the crystal-field splitting experienced by individual erbium ions.^50,65,66^ In addition, diffusion of Ga atoms into the oxide introduces excess nuclear-spin noise that broadens the optical line width and reduces the spin-coherence time of Er^3+^ ions.^67–69^ In the R–TiO_2_ films, where partial strain relaxation occurs, the broader inhomogeneous PLE line widths observed on GaAs (50 GHz) relative to GaSb (40 GHz) likely reflect differences in interfacial strain accommodation arising from the significant difference in the corresponding MCIA values. By contrast, the nearly identical line widths observed in the A–TiO_2_ films suggest that interfacial strain is not the dominant factor. Instead, static disorder associated with oxygen vacancies and defect complexes, which are more readily formed in the anatase phase, becomes the primary source of dephasing. However, the uniform doping across the film thickness employed in the samples shown in Figure 4 makes it difficult to decouple the effects of substrate-induced local strain and nuclear (Ga atoms) or electronic (O vacancies) spin noise on the REIs. A more detailed study employing spatially engineered Er^3+^ doping profiles, which allow selective probing of ions at controlled distances from the interface, is therefore required to separate these effects quantitatively and represents an important direction for future work.

## 3 Conclusion

Understanding and controlling the interplay among microstructure, interface chemistry, and optical activation is central to realizing coherent rare-earth emitters integrated with semiconductor photonic platforms. In this work, we established how substrate termination, oxygen stoichiometry, and strain collectively govern phase selection and defect formation in TiO_2_-(III–V) heterostructures. Cross-sectional STEM–EELS analysis revealed that interfacial Ga diffusion and oxygen-vacancy accumulation promote strain relaxation and rutile phase nucleation. In contrast, low-defect, arsenic-capped substrates that preserve interfacial integrity favor epitaxial anatase growth at low growth temperatures. These microscopic processes directly correlate with the optical response of Er^3+^/TiO_2_, where the balance between strain relaxation and defect-induced disorder determines the inhomogeneous line widths and optical coherence. The mechanistic insight gained here thus links growth thermodynamics to optical activation, providing a framework for engineering oxide-semiconductor heterostructures for integrated photonics.

Further improvements in film quality and stoichiometry could be achieved through precise control of plasma plume kinetics and oxygen chemical potential during growth.^46^ Introducing a diffusion barrier in tandem with arsenic capping the substrate for preserving surface integrity could help minimize interfacial pits and mitigate gallium interdiffusion. Finally, depth-selective rare-earth doping could enable targeted probing of strain and defect effects on optical coherence, providing a pathway to disentangle microscopic noise sources. Collectively, these refinements represent critical steps toward scalable, telecom-compatible, rare-earth-doped oxide films monolithically integrated with III–V semiconductors for on-chip quantum photonic technologies.

## 4 Methods

### 4.1 Target Preparation

One inch diameter undoped and 3000 ppm of Er^3+^-doped TiO_2_ targets were fabricated from TiO_2_ and Er_2_O_3_ powders (Sigma-Aldrich) by cold-pressing and high-temperature sintering (1600 °C). Full processing details and final density data are provided in Supporting Information, Section S1.2.

### 4.2 Surface and Morphology Characterization

RHEED patterns were measured in situ with an electron beam operated at 16 kV with a 1.4 mA filament current and a phosphor screen. Surface morphology was measured by tapping-mode atomic-force microscopy (Bruker Dimension Icon) equipped with a Si probe (TESPA-V2, 7 nm tip radius, 37 N/m spring constant), and the data were analyzed using Gwyddion.^70^ Crystallographic phase and orientation were identified by X-ray diffraction (Rigaku SmartLab or a four-circle Panalytical X’Pert), with peaks fit to a Voigt-function to estimate the average grain size and microstrain in the film (methods in Supporting Information, Sections S2.3 and S2.4).

### 4.3 Transmission Electron Microscopy

Cross-sectional lamellae were prepared using a Xe^+^ plasma-focused ion beam (FIB) for the R–TiO_2_ sample and a Ga^+^ liquid metal ion source FIB for the A–TiO_2_ sample (Helios 5 UXe and Helios 5 UC, respectively, Thermo Fisher Scientific). The extracted lamellae were analyzed using a probe- and image-corrected STEM (Spectra Ultra, Thermo Fisher Scientific) equipped with an X-FEG/UltiMono source operating at 300 kV accelerating voltage. Images were acquired using a 28 mrad convergence semiangle with ∼110–130 pA beam current. Acceptance angles for the ABF, LAADF, MAADF, and HAADF detectors were 0–11, 12–23, 23–44, and 49–200 mrad, respectively. HRTEM images were acquired by using parallel illumination on a Ceta-S detector. STEM-EELS data were acquired with a ContinuumK3 (Gatan) using the spectrometer’s secondary detector (fiber-optically coupled scintillator, model 1069.EXUP). Spectrum images were acquired in DualEELS mode using a 53 mrad collection semiangle and 0.3 eV/channel dispersion. Detailed lamella preparation and acquisition parameters, including beam conditions and drift-tube voltages, are provided in the Supporting Information (Section S2.7).

### 4.4 Optical Characterization

Room-temperature Raman spectroscopy measurements were performed using a dispersive Raman spectrometer (Renishaw) with a laser wavelength of λ = 514 nm and a 50× microscope objective. A long-pass filter blocked the laser and transmitted the signal with a Stokes shift of at least 180 cm^–1^ Raman shift. Cryogenic photoluminescence-excitation (PLE) spectroscopy was performed in a closed-cycle helium cryostat (Montana Instruments CryoCore) with samples cooled to <5 K. The sample was optically excited, and the fluorescence was collected using a long working distance infrared (IR) objective (Olympus LMPlan IR, 50×/0.65 NA) with a home-built confocal microscopy setup. Details of the experimental setup are provided in Supporting Information, Section S4.2.

## Supplementary Material



## Notes

Substrate/target preparation and film growth, structural and morphological characterization, interface and defect analysis, and optical and spectroscopic characterization (PDF)
