# Supplementary material for: Controlled Growth of Rare-Earth-Doped TiO2 Thin Films on III–V Semiconductors for Hybrid Quantum Photonic Interfaces
Source: ACS Appl Opt Mater. 2026 Feb 4;4(2):457–70. doi: 10.1021/acsaom.5c00585 (PMC12954842; doi:10.1021/acsaom.5c00585)
Supplement: Supplementary file 1 [file ot5c00585_si_001.pdf]

Supporting Information: Controlled growth of rare-earth-doped TiO_2 thin films on III-V semiconductors for hybrid quantum photonic interfaces

Henry C. Hammer,[†] Caleb Whittier,[‡] Nathan A. Helvy,[†] Christopher Rouleau,[¶] Nabil D. Bassim,[‡,§] and Ravitej Uppu[*,†]

[†]*Department of Physics and Astronomy, The University of Iowa, Iowa City, Iowa 52242, United States*

[‡]*Department of Materials Science and Engineering, McMaster University, Hamilton, Ontario L8S 4L7, Canada*

[¶]*Center for Nanophase Materials Sciences, Oak Ridge National Laboratory, Oak Ridge, Tennessee 37831, United States*

[§]*Canadian Centre for Electron Microscopy, McMaster University, Hamilton, Ontario L8S 4M1, Canada*

E-mail: ravitej-uppu@uiowa.edu

S1. Substrate/Target Preparation and Film Growth

S1.1 Thin Film Growth Process

Epitaxial films of (Er^{3+}:)TiO_2 were synthesized on various substrates using pulsed laser deposition (PLD). A KrF excimer laser (Coherent LPX 305F, $\lambda = 248$ nm, 25 ns pulse width) was used to ablate oxide targets. Uniform laser fluence across the illuminated target area was achieved using a projection beamline equipped with a fixed rectangular aperture, which was imaged onto the ablation targets to produce a top-hat intensity profile. The aperture size could be adjusted to tune the growth rate between $0.02 - 0.6$ Å per laser shot. Reducing the aperture size decreases the growth rate, enabling precise atomic-layer control and suppressing oxygen vacancies in oxide thin films, even when grown under high vacuum.[1] Controlled oxygen vacancy concentrations have further been used to tune lattice constants in oxide systems.[2] However, such low-growth-rate conditions result in long deposition times; for example, growth of films thicker than 50 nm require $\geq$4 h at 5 Hz laser repetition rate.

Because the present study involved a wide parameter space (substrate type, growth temperature, and dopant concentration and distribution), a moderate growth rate was chosen to enable the synthesis of multiple unique samples. The rectangular aperture was adjusted to achieve a laser fluence of 2.0 $\mathrm{J\,cm^{-2}}$, corresponding to a pulse energy of approximately 36.7 mJ and an illuminated spot size of 1.84 mm^2. Under these conditions, we measured a TiO_2 growth rate of approximately 0.17 Å per laser shot, as determined by post-growth profilometry and verified by ex situ transmission electron microscopy (TEM). With these parameters, total film growth was completed in approximately 15 min at a 5 Hz laser repetition rate. However, the total time each sample spent at the elevated temperature was approximately 1 hour, with additional time required for in-situ RHEED monitoring and sample warmup/cooldown. This throughput enabled the synthesis of several tens of unique (doped) oxide thin-film samples within a single week.

Substrate pieces (typically 5×5 mm^2 or 10×10 mm^2) within the PLD chamber, which was evacuated to a base pressure of $\approx 10^{-6}$ Torr before deposition. Growth temperatures were monitored by a pyrometer and ranged from 350 °C to 565 °C. For As-capped GaAs substrates, the substrate temperature was first raised to $\approx$ 400 °C and the surface was monitored using in-situ reflection high-energy electron diffraction (RHEED). Once the (100) GaAs RHEED pattern appeared and was assessed to be fully formed (no residual As-cap remaining), the temperature was adjusted to the desired growth temperature. For uncapped substrates, the temperature was raised to around 540 °C for GaAs ($\sim$ 500 °C for GaSb) until RHEED confirmed removal of the native oxide layer, followed by adjustment to the target growth temperature T_{grow}. Across the substrate, temperature variations were typically within ± 10 °C. During growth, both the substrate holder and target carousel were rotated to ensure uniform film deposition.

Table S1: Growth parameters of undoped TiO_2-(III-V) samples. The buffer layer was TiO_2 in all cases grown under high vacuum (10^{-6} Torr).

**Sample**	**Substrate**	**Buffer Shots**	$\mathbf{T_{buf}}$ (°C)	**Growth Shots**	$\mathbf{T_{grow}}$ (°C)
GaAs-HT-1	GaAs	260	380	500	380
GaAs-HT-2	GaAs	450	565	5000	575
GaAs-HT-7	GaAs	350	450	3000	450
GaAs-HT-8	GaAs	450	540	5000	550/440 (start/end)
GaAs-HT-9	GaAs	470	550	5000	555/510 (start/end)
GaAs-HT-10	GaAs	500	525	5000	530/440 (start/end)
GaSb-HT-2	GaSb	450	500	2000	500/490 (start/end)
GaSb-HT-3	GaSb	650	520	5000	520

Oxide growth was initiated under high vacuum by manually firing laser shots until the RHEED pattern changed from the substrate to the film (typically 100 laser pulses). When the lattice mismatch between the substrate and target was significant, an alternate material buffer layer (about 1-2 nm) was used (e.g., CeO_2 buffer was used for growing TiO_2 on Si). If the lattice match between the substrate and target crystals was not close enough to warrant direct growth of the target on the substrate, such as with TiO_2 on Si, we used a different

Table S2: Growth parameters of Er^{3+} doped TiO_2-(III-V) samples. The buffer layer was grown under high vacuum (10^{-6} Torr), while the film was grown at an oxygen pressure of 20 mTorr.
Legend– T: TiO_2; E: Er^{3+}:TiO_2; C: CeO_2.

**Sample**	**Substrate**	**Buffer**	**Buffer Shots**	$\mathbf{T_{buf}}$ (°C)	**Thin film**	**Growth Shots**	$\mathbf{T_{grow}}$ (°C)
GaAs-HT-3	GaAs	T	470	540	T/E/T	2350/500/2500	555/440 (start/end)
GaAs-HT-4	GaAs	T/E	120/350	540	E	5000	550/440 (start/end)
GaAs-HT-5	GaAs	T	470	545	T/E/T	2350/500/2500	545/495 (start/end)
GaAs-HT-6	GaAs	T	470	540	T/E/T	2000/500/2500	550
GaAs-LT-1	GaAs	T	70	370	T/E	500/2500	370
GaAs-LT-2	GaAs	T	70	390	T/E/T	180/100/2650	390
GaAs-LT-3	GaAs	T	70	390	T/E/T	780/100/2050	390
GaAs-LT-4	GaAs	T	120	400	T/E	500/2500	400
GaAs-LT-5	GaAs	T	120	350	T/E	500/2500	350
GaAs-LT-6	GaAs	T	200/300	350/390	T/E	500/2500	390
GaAs-LT-7	GaAs	T	70	390	T/E/T	1380/100/1450	390
GaAs-LT-8	GaAs	T	70	390	T/E/T	1980/100/850	390
GaAs-LT-9	GaAs	T	70	390	T/E/T	1980/100/850	390
GaAs-LT-10	GaAs	T	120	390	T/E/T	1930/100/850	390
GaAs-LT-11	GaAs	T	70	390	T/E	430/2500	390
GaAs-LT-12	GaAs	T	70	390	T/E	430/2500	390
GaAs-LT-13	GaAs	T	70	390	T/E/T	2580/100/250	390
GaSb-HT-1	GaSb	T/E	200/500	500	E	5000	480/430 (start/end)
GaSb-LT-1	GaSb	T	101	370	T/E	399/2500	370
Si-HT-1	Si	T/E	80/40	490	E	2500	500
Si-HT-2	Si	C	70	500	T/E	430/2500	500
Si-HT-3	Si	C	200	500	T/E	300/2500	500
Si-HT-4	Si	C	170	500	T/E/T	680/100/2050	500
Si-HT-5	Si	C	170	500	T/E/T	1880/100/850	500
Si-LT-1	Si	T	90	350	T/E	500/2500	365
R-LT-1	R-TiO_2(110)	T	70	400	T/E	500/2500	400

material (i.e., CeO_2) as a buffer layer. Subsequently, the deposition target was switched to (Er^{3+}:)TiO_2. Detailed sample growth conditions are presented in Tables S1, S2.

After this initial deposition, oxygen was introduced by opening the mass flow controller to raise the chamber pressure to 20 mTorr while the deposition was paused. Once pressure stabilized, additional laser pulses were fired until the desired film thickness was achieved, approximately 60 nm (∼3000 total shots; A-TiO_2) or 90 nm (∼5300 total shots; R-TiO_2). Following deposition, samples were annealed in situ for approximately 30 min during cool down. At a substrate heater temperature of ∼ 200 °C, the chamber was vented to atmospheric pressure and samples were removed. The samples were allowed to cool to room temperature before further characterization.

S1.2 Target Preparation

The undoped TiO_2 target used as a buffer or undoped layer during growth was identical to that described by Bell et al.[3] It was prepared by cold-pressing TiO_2 powder into a pellet under a pressure of 65 MPa using a hydraulic press (6 ton press with a 1" target), followed by sintering at 1400 °C for 6 h.

The Er^{3+}:TiO_2 target was made fabricated as follows. First, 106 mg of Er_2O_3 powder (Sigma-Aldrich) was thoroughly crushed and mixed with 12.07 g of TiO_2 powder, corresponding to a nominal Er^{3+} concentration of approximately 3600 ppm (0.36%). From this mixture, 9.99 g was compacted in a hydraulic pellet press into a 1" diameter disc. Subsequently, it was crushed in a ball mill and approximately 7 g of the recrushed mixture was recompacted for 3 h to create a 1" diameter disc of 6 mm thickness. The resulting pellet was then sintered at 1600 °C for 16 h.

The sintered pellet was characterized to estimate the density as summarized in Table S3. Given the low (75%) density compared to the expected value for rutile phase TiO_2, the pellet was crushed again, re-compacted, and sintered at 1600 °C for 36 h. After completing the full preparation sequence, the resulting target pellet achieved a relative density of ≥80%,

which was considered sufficient for use in subsequent thin-film growth experiments.

Table S3: Parameters of the Er^{3+}:TiO_2 target after each bake during development.

**Parameter**	**Post Bake 1**	**Post Bake 2**
Bake Temperature (°C)	1600	1600
Bake Time (h)	16	36
Diameter (inches)	0.888	0.930
Thickness (inches)	0.217	0.185
Weight (g)	7.06	7.03
Volume (cm^3)	2.12	2.06
Density ($g\,cm^{-3}$)	3.20	3.42
Density† (%)	≥75%	≥80%

† Lower bound of the target density relative to that of pure rutile $\rho = 4.23$ $g\,cm^{-3}$.

S2. Structural and Morphological Characterization

S2.1 Additional AFM Data

For completeness, we include further AFM data within this subsection. In total, the surface morphology of 32 samples were analyzed. This includes arsenic-capped GaAs (12), uncapped GaAs (10), uncapped GaSb (4), and silicon-on-insulator (SOI, 6) substrates. The surface RMS roughness statistics for each substrate is included in Fig. S1(a). Similar to uncapped GaAs substrates, samples with uncapped GaSb substrates produce consistently rougher surfaces in comparison to arsenic-capped GaAs substrates. Among the TiO_2 thin films grown on SOI substrates, we observed that the addition of a CeO_2 buffer at a growth temperature of 500 °C improved smoothness, although not with the same consistency as with arsenic-capped GaAs. Also note that we did not observe a strict correlation between the film RMS roughness and growth temperature as evident in Figure S1(b) for any substrate, in contrast to previous studies on TiO_2 thins film grown on Si(100) substrates using atomic layer deposition.[4]

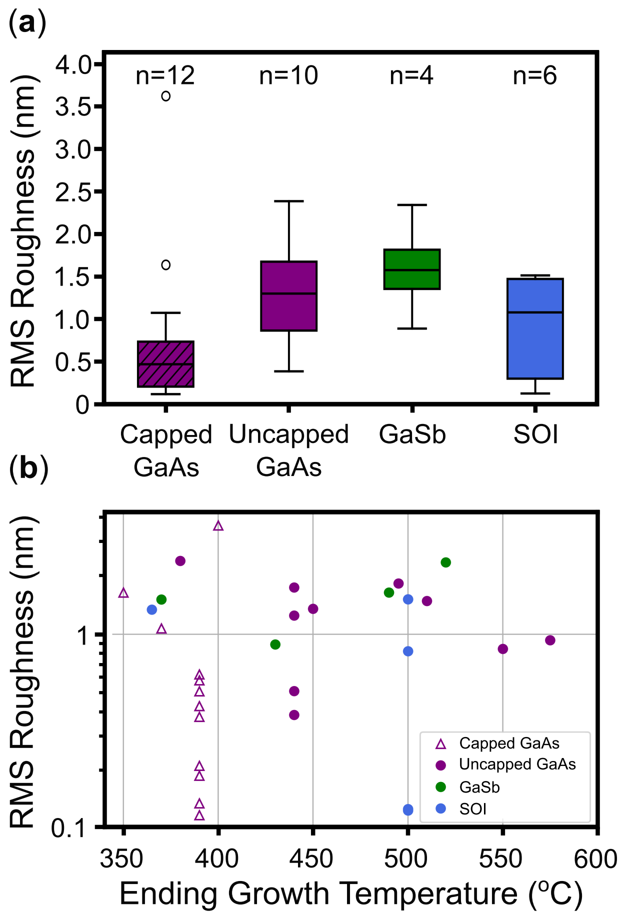

Figure S1: (a) Box-and-whisker plot of mean RMS roughness values extracted across multiple scans organized by substrate. The number ('n') of unique samples utilized in each substrate's statistics are included within the plot. (b) Scatter plot comparing ending growth temperature (T_{grow}) to mean RMS roughness value extracted across multiple scans.

S2.2 Minimal Co-Incident Interface Area Calculation

The algorithm used to compute the MCIA (minimum coincident interface area) for R-TiO_2 and A-TiO_2 is based on the approach of Zur & McGill.[5] Each substrate and thin-film orientation is defined by a crystal plane with Miller indices (hkl) perpendicular to the growth direction. For a given orientation, lattice vectors **a** and **b** are constructed such that they (1) lie within the specified crystal plane and (2) accurately describe the translational symmetry of the lattice within that plane. An integer cut-off parameter, $n_{\max}$, is then chosen to define the maximum superlattice area considered for both the substrate and oxide crystals in the

MCIA computation. The superlattice area is defined as $A_{sc} = nA_{\text{prim}}$, where A_{prim} is the area of the primitive lattice unit cell in the specified plane, bounded by the coordinates $(\mathbf{0}, \mathbf{a}, \mathbf{b}, \mathbf{a}+\mathbf{b})$. In our calculations, we used n_{max}=200, which yielded approximately 30,000 non-unique superlattice matching configurations, with A_{sc} values up to $3,000$ Å^2 evaluated for substrate and oxide thin film.

It has been shown that a set of primitive translations ($\mathbf{u}$,$\mathbf{v}$) for a superlattice can be generated using a simple matrix equation:[6]

$$\begin{bmatrix} \mathbf{u} \\ \mathbf{v} \end{bmatrix} = \begin{bmatrix} i & j \\ 0 & m \end{bmatrix} \begin{bmatrix} \mathbf{a} \\ \mathbf{b} \end{bmatrix} \tag{1}$$

where, $i \cdot m = n$ with $i, m \in \mathbb{Z}^+$ and $0 \leq j \leq m - 1$.

Using these relations, we compute all superlattice configurations for $2 \leq n \leq n_{\text{max}}$ for both the substrate and thin film. These configurations are then reduced to retain only unique ($\mathbf{u}$,$\mathbf{v}$) pairs:

1. If $\mathbf{u} \cdot \mathbf{v} < 0$, replace $\mathbf{b}$ with -$\mathbf{b}$. Proceed to step 2.

2. If $|\mathbf{u}| > |\mathbf{v}|$, interchange $\mathbf{u}$ with $\mathbf{v}$ and return to step 1; otherwise, proceed to step 3.

3. If $|\mathbf{v}| > |\mathbf{v}+\mathbf{u}|$, replace $\mathbf{v}$ with $\mathbf{v}+\mathbf{u}$ and return to step 1; otherwise, proceed to step 4.

4. If $|\mathbf{v}| > |\mathbf{v}-\mathbf{u}|$, replace $\mathbf{v}$ with $\mathbf{v}-\mathbf{u}$ and return to step 1; otherwise, the reduction is complete.

After running the reduction algorithm for each set of superlattice vectors at a given n, the reduced superlattice parameters are calculated: $|\mathbf{u}|$, $|\mathbf{v}|$, A_{sc} and the angle α=$\cos^{-1}(\frac{|\mathbf{u}\cdot\mathbf{v}|}{|\mathbf{u}||\mathbf{v}|})$ between $\mathbf{u}$ and $\mathbf{v}$. These reduced superlattice parameters of the substrate and oxide are then compared to identify the most likely epitaxial growth orientation. An interface is designated as lattice-matched when all reduced parameters ($X = |\mathbf{u}|, |\mathbf{v}|, A_{sc}, \alpha$) of the substrate and

film satisfy the following condition:

$$\frac{X_{\text{sub}} - X_{\text{film}}}{X_{\text{film}}} < 0.01 \tag{2}$$

We selected the 1% threshold for data presented in Fig. 6 of the main text (see Table S4) to account for previously observed polycrystalline R-TiO_2, which exhibited low but non-negligible microstrain levels.[4,7] We also include 1% threshold MCIA results for brookite phase (B-TiO_2) on various substrates to account for potential multiphase synthesis during growth (see Table S5). Modifying the threshold can significantly affect the calculated MCIA, epitaxial condition, and lattice mismatch values. In general, increasing the threshold values decreases the MCIA while increasing the lattice mismatch, and vice versa. To illustrate this relationship, Table S6 includes additional MCIA values calculated using a 2% threshold.

We have also included the MCIA results for fluorite (cubic) CeO_2 on Si in Table S7 and rutile and anatase TiO_2 in Table S8.

Table S4: Results for <1% threshold MCIA calculations of R-TiO_2 and A-TiO_2 with substrates utilized in growths, complementing the data in the results computed with <1% threshold in Fig. 6 of the main text.

**Film matching face (hkl)**	**Epitaxial Condition**	**MCIA** ($\AA^2$)	**Epitaxial Condition**	**MCIA** ($\AA^2$)
	Rutile		Anatase	
GaAs (100)				
(100)	GaAs$[01\bar{3}]$ $\parallel$ R[001]	1352	GaAs[010] $\parallel$ A$[0\bar{1}0]$	703
(001)	GaAs$[0\bar{1}\bar{1}]$ $\parallel$ R[160]	782	GaAs$[0\bar{1}\bar{1}]$ $\parallel$ A$[\bar{1}10]$	64
(110)	GaAs$[01\bar{3}]$ $\parallel$ R[001]	637	GaAs$[01\bar{1}]$ $\parallel$ A$[\bar{1}10]$	383
(101)	N/A	>3000	N/A	>3000
(111)	N/A	>3000	N/A	>3000
(210)	GaAs$[0\bar{3}\bar{1}]$ $\parallel$ R$[1\bar{2}0]$	639	GaAs$[00\bar{1}]$ $\parallel$ A$[1\bar{2}0]$	481
GaSb (100)				
(100)	GaSb$[00\bar{1}]$ $\parallel$ R[010]	1679	GaSb$[01\bar{1}]$ $\parallel$ A$[0\bar{1}0]$	1299
(001)	GaSb$[0\bar{1}\bar{1}]$ $\parallel$ R[110]	168	GaSb$[0\bar{1}\bar{3}]$ $\parallel$ A$[\bar{5}10]$	186
(110)	GaSb$[01\bar{1}]$ $\parallel$ R$[\bar{1}10]$	501	GaSb$[01\bar{1}]$ $\parallel$ A$[\bar{1}10]$	1020
(101)	N/A	>3000	N/A	>3000
(111)	N/A	>3000	N/A	>3000
(210)	GaSb$[01\bar{5}]$ $\parallel$ R$[1\bar{2}0]$	1924	N/A	>3000
Si (100)				
(100)	Si[051] $\parallel$ R[010]	573	Si$[00\bar{1}]$ $\parallel$ A[010]	1443
(001)	Si[051] $\parallel$ R[010]	382	Si[031] $\parallel$ A$[\bar{8}10]$	294
(110)	Si$[01\bar{1}]$ $\parallel$ R$[\bar{1}10]$	1060	Si$[01\bar{1}]$ $\parallel$ A[001]	663
(101)	N/A	>3000	N/A	>3000
(111)	N/A	>3000	N/A	>3000
(210)	Si$[01\bar{1}]$ $\parallel$ R$[00\bar{1}]$	823	Si$[01\bar{1}]$ $\parallel$ A[001]	1616

Table S5: Results for <1% threshold MCIA calculations of brookite TiO_2 (B) with substrates utilized in growths.

**Film matching face (hkl)**	**Epitaxial Condition**	**MCIA** ($\AA^2$)
GaAs(100)/B(010)	GaAs$[0\bar{1}\bar{3}]$ $\parallel$ B[001]	320
GaSb(100)/B(010)	GaSb$[01\bar{5}]$ $\parallel$ B$[\bar{1}00]$	482
Si(100)/B(010)	Si$[01\bar{1}]$ $\parallel$ B$[\bar{1}00]$	412
R(100)/B(010)	R[001] $\parallel$ B[001]	366
R(001)/B(010)	R[210] $\parallel$ B$[\bar{1}00]$	1053
R(110)/B(010)	R[001] $\parallel$ B[001]	637
A(001)/B(010)	A$[\bar{2}10]$ $\parallel$ B[001]	642
CeO2(100)/B(010)	CeO2$[01\bar{1}]$ $\parallel$ B$[\bar{1}00]$	411
CeO2(110)/B(010)	CeO2$[1\bar{1}0]$ $\parallel$ B$[\bar{1}00]$	1163
CeO2(111)/B(010)	CeO2$[2\bar{1}\bar{1}]$ $\parallel$ B[001]	962

Table S6: Results for <2% threshold MCIA calculations of R-TiO_2 and A-TiO_2 with substrates utilized in growths, complementing the data in the results computed with <1% threshold in Fig. 6 of the main text.

**Film matching face (hkl)**	**Epitaxial Condition**	**MCIA** (Å^2)	**Epitaxial Condition**	**MCIA** (Å^2)
	Rutile		Anatase	
GaAs (100)				
(100)	GaAs[01$\bar{1}$] || R[001]	382	GaAs[010] || A[0$\bar{1}$0]	322
(001)	GaAs[05$\bar{1}$] || R[130]	209	GaAs[0$\bar{1}\bar{1}$] || A[$\bar{1}$10]	64
(110)	GaAs[01$\bar{1}$] || R[001]	384	GaAs[01$\bar{1}$] || A[$\bar{1}$10]	383
(101)	N/A	>3000	N/A	>3000
(111)	N/A	>3000	N/A	>3000
(210)	GaAs[01$\bar{3}$] || R[00$\bar{1}$]	639	GaAs[00$\bar{1}$] || A[1$\bar{2}$0]	481
GaSb (100)				
(100)	GaSb[01$\bar{2}$] || R[010]	933	GaSb[01$\bar{3}$] || A[001]	649
(001)	GaSb[0$\bar{1}\bar{1}$] || R[110]	168	GaSb[01$\bar{3}$] || A[$\bar{5}$10]	186
(110)	GaSb[01$\bar{1}$] || R[$\bar{1}$10]	387	GaSb[01$\bar{3}$] || A[001]	279
(101)	N/A	>3000	N/A	>3000
(111)	N/A	>3000	N/A	>3000
(210)	GaSb[051] || R[1$\bar{2}$0]	1454	GaSb[01$\bar{3}$] || A[00$\bar{1}$]	647
Si (100)				
(100)	Si[051] || R[010]	573	Si[01$\bar{1}$] || A[0$\bar{1}$0]	162
(001)	Si[051] || R[010]	382	Si[01$\bar{1}$] || A[$\bar{1}$00]	236
(110)	Si[01$\bar{1}$] || R[$\bar{1}$10]	518	Si[00$\bar{1}$] || A[$\bar{1}$10]	205
(101)	N/A	>3000	N/A	>3000
(111)	N/A	>3000	N/A	>3000
(210)	Si[01$\bar{1}$] || R[00$\bar{1}$]	823	Si[01$\bar{1}$] || A[001]	962

Table S7: Results for <1% threshold MCIA calculations of fluorite (cubic) CeO_2 with Si.

**Film matching face (hkl)**	**Epitaxial Condition**	**MCIA** (Å^2)
Si(100)/CeO_2(100)	Si[01$\bar{1}$] || CeO_2[01$\bar{1}$]	29
Si(100)/CeO_2(110)	Si[01$\bar{1}$] || CeO_2[1$\bar{1}$0]	103
Si(100)/CeO_2(111)	Si[01$\bar{1}$] || CeO_2[01$\bar{1}$]	532

Table S8: Results for <1% threshold MCIA calculations of fluorite (cubic) CeO_2 (C) with R-TiO_2 (R) and A-TiO_2 (A).

**Matching faces (hkl)**	**Epitaxial Condition**	**MCIA ($Å^2$)**	**Epitaxial Condition**	**MCIA ($Å^2$)**
	Rutile		Anatase	
GaAs (100)				
(100)/C(100)	R[010] || $CeO_2[01\bar{1}]$	613	A[$0\bar{1}0$] || $CeO_2[00\bar{1}]$	1437
(100)/C(110)	R[010] || $CeO_2[1\bar{1}0]$	746	A[001] || $CeO_2[1\bar{1}0]$	722
(100)/C(111)	R[010] || $CeO_2[0\bar{1}1]$	610	A[001] || $CeO_2[0\bar{1}1]$	505
(001)/C(100)	R[100] || $CeO_2[05\bar{1}]$	190	A[$\bar{8}10$] || $CeO_2[031]$	293
(001)/C(110)	R[$4\bar{1}0$] || $CeO_2[1\bar{1}0]$	721	A[$\bar{6}10$] || $CeO_2[1\bar{1}\bar{4}]$	499
(001)/C(111)	R[010] || $CeO_2[01\bar{1}]$	1060	A[$\bar{1}\bar{1}0$] || $CeO_2[2\bar{1}\bar{1}]$	356
(110)/C(100)	R[$\bar{1}10$] || $CeO_2[00\bar{1}]$	1056	A[001] || $CeO_2[01\bar{1}]$	511
(110)/C(110)	R[001] || $CeO_2[1\bar{1}0]$	867	A[$\bar{1}10$] || $CeO_2[1\bar{1}0]$	1016
(110)/C(111)	R[001] || $CeO_2[2\bar{1}\bar{1}]$	1214	A[$\bar{1}10$] || $CeO_2[2\bar{1}\bar{1}]$	254
(101)/C(100)	N/A	>3000	N/A	>3000
(101)/C(110)	N/A	>3000	N/A	>3000
(101)/C(111)	N/A	>3000	N/A	>3000
(111)/C(100)	N/A	>3000	N/A	>3000
(111)/C(110)	N/A	>3000	N/A	>3000
(111)/C(111)	N/A	>3000	N/A	>3000
(210)/C(100)	R[$00\bar{1}$] || $CeO_2[01\bar{1}]$	820	A[001] || $CeO_2[01\bar{1}]$	1610
(210)/C(110)	R[$1\bar{2}0$] || $CeO_2[1\bar{1}0]$	998	A[$00\bar{1}$] || $CeO_2[1\bar{1}0]$	2581
(210)/C(111)	R[$1\bar{2}0$] || $CeO_2[01\bar{1}]$	1825	N/A	>3000

S2.3 XRD System Parameters

Table S9 summarizes the key parameters for each XRD system used in this work. Typical θ–2θ scan step sizes ranged from 0.01° to 0.05°, with scan rates of approximately 5°/min. For specific measurements collected on the Rigaku system (e.g., Fig. 7 in the main text), an additional 0.5° horizontal entrance slit was employed together with the 5° Soller slit. Under these conditions, the detector operated in "0D" mode rather than the "1D" (line) mode used for all other measurements.

Table S9: XRD θ-2θ measurement parameters utilized for characterizing the microstructural properties of the grown oxide thin films.

**Parameter**	**Rigaku Smartlab**	**Panalytical X'Pert**
X-Ray Source	$CuK_{\alpha1}$ ($CuK_{\alpha2}$)	$CuK_{\alpha1}$ ($CuK_{\alpha2}$)
X-Ray Wavelength	1.54059 Å (1.54441 Å)	1.54059 Å (1.54441 Å)
Voltage	40 kV	45 kV
Current	44 mA	40 mA
Incident Slit	2.0 mm	0.05 mm
Receiving Slit 1	3.0 mm	0.01 mm
Receiving Slit 2	3.0 mm	N/A
Length Limiting Slit	2.0 mm	N/A
CuK_{β} Filter	Ni foil	Ni beam attenuator (0.125 mm height)
Monochromator	N/A	Hybrid 2xGe(220)
Soller Slit	5°	2.29°

S2.4 XRD Data Fitting: Grainsize & Microstrain Analysis

Average grain size and microstrain were determined from the θ–2θ scans by separating the Gaussian and Lorentzian contributions to the peak linewidth. Each measured spectrum was normalized to its maximum intensity and fit with the convolution of (1) a Voigt function and (2) an instrument response function, as illustrated in Fig. S2. The instrument response was obtained by performing a θ–2θ scan on a (110)- or (100)-cut rutile TiO_2 wafer, corresponding to thin films grown on GaAs and GaSb substrates, respectively. The response function was normalized to the integrated counts over the 2θ range used for convolution during the fitting

process. All θ–2θ scans of both sample and reference wafers were acquired using identical instrumental settings.

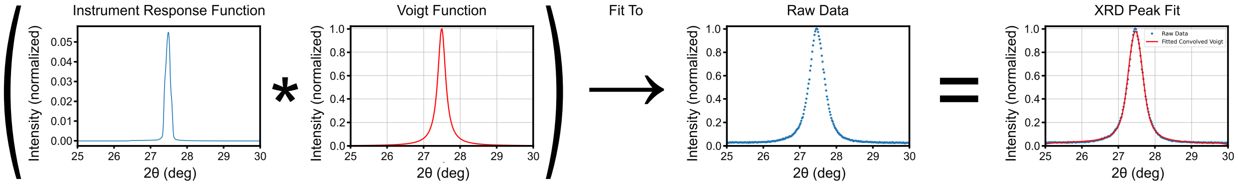

Figure S2: Diagram of the fitting procedure utilized to extract the average grain size (τ) and microstrain (ϵ).

Grain size (τ) and microstrain (ϵ) were calculated using the Scherrer and Wilson equations, respectively, with $\lambda = 1.54059$ Å (Cu $K_{\alpha 1}$). Although both Cu $K_{\alpha 1}$ and Cu $K_{\alpha 2}$ radiation were present, their individual contributions to the TiO_2 peaks could not be resolved due to limited signal-to-noise ratio (SNR). Consequently, using either wavelength yielded τ and ϵ values that agreed within fitting error. While the SNR of spectra collected on the Rigaku system could be modestly improved through rebinning, filtering, or increasing the slit width, we chose to retain the unprocessed data because the extracted linewidths were consistent with those measured on the X'Pert system, which employs a hybrid 2-bounce Ge(110) monochromator.

For completeness, the error propagation for τ and ϵ is outlined below. The Lorentzian and Gaussian linewidths (Γ_L, Γ_G) and their associated uncertainties (σ_{Γ_L}, σ_{Γ_G}) were derived from the Voigt fit as:

$$\Gamma_L = 2\gamma, \qquad \sigma_{\Gamma_L} = 2\sigma_\gamma, \tag{1a}$$

$$\Gamma_G = 2\sqrt{2\ln 2}\,\sigma, \qquad \sigma_{\Gamma_G} = 2\sqrt{2\ln 2}\,\sigma_\sigma. \tag{1b}$$

These values are then used to calculate the Voigt linewidth (Γ_V) and its error (σ_{Γ_V}):

$$\tilde{\sigma}_{\Gamma_{\mathrm{L}}} = \sigma_{\Gamma_{\mathrm{L}}}\Big(0.5346 + \frac{0.2166\Gamma_{\mathrm{L}}}{\sqrt{0.2166(\Gamma_{\mathrm{L}})^2 + (\Gamma_{\mathrm{G}})^2}}\Big) \tag{4a}$$

$$\tilde{\sigma}_{\Gamma_{\mathrm{G}}} = \sigma_{\Gamma_{\mathrm{G}}}\Big(\frac{\Gamma_{\mathrm{G}}}{\sqrt{0.2166(\Gamma_{\mathrm{L}})^2 + (\Gamma_{\mathrm{G}})^2}}\Big) \tag{4b}$$

$$\sigma_{\Gamma_{\mathrm{V}}} = \sqrt{(\tilde{\sigma}_{\Gamma_{\mathrm{L}}})^2 + (\tilde{\sigma}_{\Gamma_{\mathrm{G}}})^2} \tag{4c}$$

Finally, τ and ϵ are extracted and their errors (σ_τ and σ_ϵ) are propagated according to:

$$\sigma_\tau = \tau\sqrt{\Big(\frac{\sigma_{\Gamma_{\mathrm{L}}}}{\Gamma_{\mathrm{L}}}\Big)^2 + \Big(\frac{\sigma_{2\theta_{(hkl)}}}{2} \cdot \tan(\theta_{(hkl)})\Big)^2} \tag{5a}$$

$$\sigma_\epsilon = \epsilon\sqrt{\Big(\frac{\sigma_{\Gamma_{\mathrm{G}}}}{\Gamma_{\mathrm{G}}}\Big)^2 + \Big(\frac{\sigma_{2\theta_{(hkl)}}}{2} \cdot \frac{1}{\cos(\theta_{(hkl)})\sin(\theta_{(hkl)})}\Big)^2} \tag{5b}$$

S2.5 XRD Data

We present the extracted values of grain size τ and microstrain ϵ, along with corresponding linewidth components, for samples whose XRD data could be adequately fit. All values were derived using the $\mathrm{CuK}_{\alpha 1}$ source wavelength of $\lambda = 1.54059$ Å. Table S10 lists the results for the R-TiO_2 (110) reflection obtained from samples grown on GaAs substrates, measured both without and with an additional 0.5° entrance horizontal slit (used in conjunction with a 5° Soller slit).

To complement the discussion of R-TiO_2 films grown on GaAs, we discuss the θ-2θ XRD scan of a TiO_2 grown on oxide-desorbed GaSb grown at 490 °C, shown in Fig. S3. The diffraction pattern exhibits its most intense reflection near the R-TiO_2 (200) position (consistent across all samples), rather than the R(002) orientation predicted by the lowest MCIA value. The absence of a clear R(002) peak may result from overlap with the intense GaSb(400) substrate reflection, slight substrate miscut, or orientation bias introduced during

Table S10: Voigt fit parameters for the XRD (110) peak of R-TiO_2 for samples grown on GaAs substrates, from which microstrain (ϵ) and grain size (τ) were extracted.

**Sample**	**XRD System**	**2θ (°)**	Γ_V (°)	Γ_G (°)	Γ_L (°)	ϵ **(%)**	τ (nm)
Data collected with a 5° Soller slit.							
GaAs-HT-2†	Rigaku	27.494(1)	0.26(1)	0.000(1)	0.260(5)	0.000(1)	31.4(6)
GaAs-HT-2	X'Pert	27.399(4)	0.63(6)	0.37(5)	0.40(5)	0.66(9)	21(3)
GaAs-HT-8	X'Pert	27.452(5)	0.73(7)	0.43(6)	0.47(6)	0.8(1)	17(2)
GaAs-HT-4	X'Pert	27.323(5)	0.56(7)	0.29(6)	0.41(6)	0.5(1)	20(3)
GaAs-HT-9	X'Pert	27.422(2)	0.68(2)	0.44(2)	0.38(2)	0.78(3)	21(1)
GaAs-HT-10	X'Pert	27.483(6)	0.53(8)	0.37(6)	0.27(7)	0.7(1)	31(8)
GaAs-HT-6	X'Pert	27.433(6)	0.45(8)	0.21(8)	0.35(6)	0.4(1)	23(4)
GaAs-HT-5	X'Pert	27.48(1)	0.6(1)	0.3(1)	0.4(1)	0.5(2)	19(5)
GaAs-HT-3	X'Pert	27.50(1)	0.8(2)	0.6(1)	0.2(2)	1.1(2)	39(32)
GaAs-HT-7	Rigaku	27.459(9)	0.4(1)	0.1(2)	0.39(8)	0.2(4)	21(4)
GaAs-LT-4	Rigaku	27.45(2)	0.7(3)	0.2(3)	0.6(2)	0.4(6)	14(4)
Additional 0.5° entrance slit together with the 5° Soller slit.							
GaAs-HT-2*	Rigaku	27.399(2)	0.61(2)	0.38(2)	0.37(2)	0.68(4)	22(1)
GaAs-HT-8	Rigaku	27.433(2)	0.81(3)	0.54(3)	0.44(3)	0.96(5)	19(1)
GaAs-HT-4	Rigaku	27.379(4)	0.61(5)	0.26(6)	0.49(4)	0.47(9)	17(1)
GaAs-HT-9	Rigaku	27.422(1)	0.67(2)	0.43(2)	0.40(2)	0.76(3)	21(1)
GaAs-HT-10	Rigaku	27.407(4)	0.64(6)	0.37(5)	0.41(5)	0.67(9)	20(2)
GaAs-HT-6	Rigaku	27.363(5)	0.50(6)	0.35(5)	0.26(5)	0.62(9)	32(7)
GaAs-HT-3	Rigaku	27.407(2)	0.68(3)	0.49(2)	0.32(3)	0.87(4)	26(2)

†10 mm receiving slits utilized instead of 3 mm.
*Appears in Figure 7(a),(b) of main text.

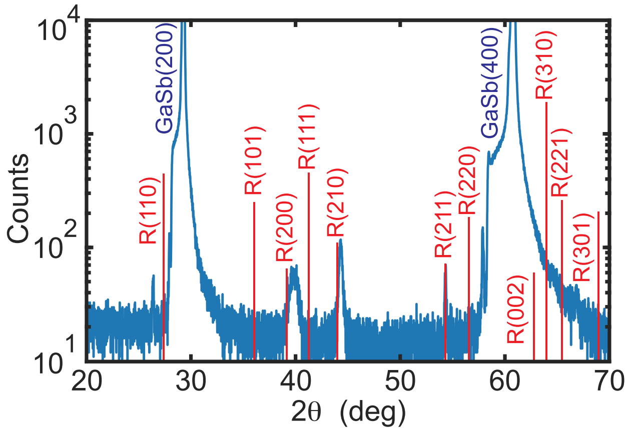

Figure S3: θ-2θ XRD spectra for sample GaSb-HT-2. Vertical red lines indicate the expected R-TiO_2 peak positions of a perfect crystal using a $CuK_{\alpha 1}$ x-ray source.

early nucleation. The observed feature at R(210) could originate from scattering from the sample holder rather than a true crystallographic reflection due to the small (5×5 mm^2) sample size. Typical values of 2θ for the R(200) peak ranged from $39.47^o - 39.81^o$ (see Table S11). Using Scherrer and Wilson equations from fitting the R(200) peak, we extract $\epsilon = 1.1 - 1.4\%$, indicating compressively strained films. Within fitting error, the peak was purely Gaussian, resulting in an unresolved grain size τ. The results for the R-TiO_2 (200) reflection from all samples grown on GaSb substrate are summarized in Table S11.

Table S11: Voigt fit parameters for the XRD (200) peak of R-TiO_2, from which microstrain (ϵ) and grain size (τ) were extracted. Data was collected with a 5° Soller slit.

**Sample**	**XRD System**	2θ (°)	Γ_V (°)	Γ_G (°)	Γ_L (°)	ϵ (%)	τ (nm)
GaSb-HT-2†	Rigaku	39.81(1)	0.9(2)	0.9(2)	0.0(3)	1.1(2)	N/A
GaSb-HT-2*	Rigaku	39.74(1)	0.9(1)	0.9(1)	0.0(2)	1.1(1)	N/A
GaSb-HT-1	Rigaku	39.47(4)	1.2(7)	1.2(5)	0.0(9)	1.4(6)	N/A

†10 mm receiving slits utilized instead of 3 mm.
*Shown in Fig. S3.

Finally, the A-TiO_2 (004) reflection from a thin film grown at low temperature on GaSb was reported in Fig. 7(c),(d) of the main text. Other low-temperature samples exhibiting the anatase phase (as confirmed by Raman spectroscopy) did not show detectable diffraction peaks under various Rigaku XRD configurations (e.g., receiving slit width, integration time,

etc.). We attribute this to the combination of the films' relatively small thickness ($\sim$60 nm) and the intrinsically weak diffraction intensity of the anatase (004) reflection, rather than to a lack of crystallinity.

S2.6 GIXRD Measurement

The grazing incidence X-ray diffraction (GIXRD) measurement of an A-TiO_2 thin film (sample GaAs-LT-3) was performed using a fixed incident angle of $\omega = 0.3°$, which is above the critical acceptance angle for A-TiO_2 of $\theta_c \approx 0.275$ °(0.276 °) for Cu$K_{\alpha 1}$ (Cu$K_{\alpha 2}$) radiation for an A-TiO_2 film with a density of 3.78 $\mathrm{g\,cm^{-3}}$.[8] Instrumental parameters for the GIXRD measurements are listed in Table S12.

A weak, asymmetric reflection was observed near the anatase (101) position as shown in Fig. S4. A double-Voigt fit accurately reproduced the lineshape, capturing both the residual Cu $K_{\alpha 1,\alpha 2}$ splitting and instrumental broadening. The peak position agrees with the expected anatase (101) d-spacing within fitting uncertainty, while its relatively low intensity reflects the film's small thickness and the grazing-incidence geometry. The primary Voigt component (approximating Cu $K_{\alpha 1}$) was centered at $2\theta = 25.661(6)°$, with a total linewidth $\Gamma_V = 0.6(2)°$, composed of Gaussian and Lorentzian contributions of $\Gamma_G = 0.4(1)°$ and $\Gamma_L = 0.2(3)°$, respectively. From this fit, the microstrain and average grain size were estimated as $\epsilon = 0.8(2)\%$ and $\tau = 36(40)$ nm.

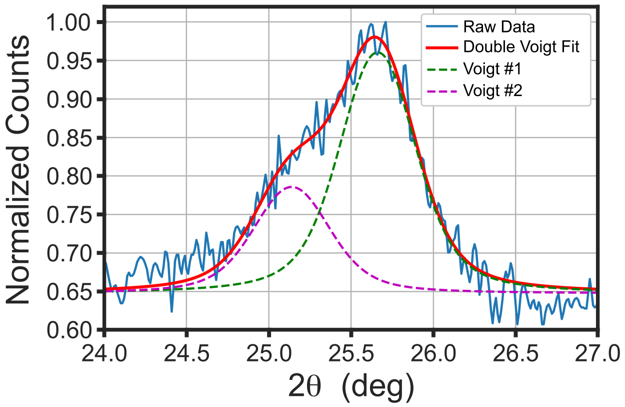

Figure S4: GIXRD measurement and resulting fit for sample GaAs-LT-3.

Table S12: GIXRD measurement parameters utilized for characterizing the A-TiO_2 thin film structure at fixed incident angle $\omega = 0.3°$.

**Parameter**	**Rigaku XRD System**
X-Ray Source	$CuK_{\alpha 1}$ ($CuK_{\alpha 2}$)
X-Ray Wavelength	1.54059 Å (1.54441 Å)
Incident Angle (ω)	0.3 °
Scan Speed	0.2 ° min^{-1}
Step Size	0.01 °
Voltage	40 kV
Current	44 mA
Incident Slit	2.0 mm
Receiving Slit 1	3.0 mm
Receiving Slit 2	3.0 mm
Length Limiting Slit	2.0 mm
CuK_β Filter	Ni foil
Monochromator	N/A
Soller Slit (Entrance & Exit)	5 °

S2.7 Lamella Preparation and STEM Analysis Details

The lamellae were prepared for scanning transmission electron microscopy (STEM) analysis using focused ion beam (FIB). The R-TiO_2 sample was prepared in a Helios 5 UXe (Thermo Fisher Scientific) equipped with an inductively coupled plasma Xe^+ source. A protective layer of carbon was first placed via electron-beam induced deposition (EBID), followed by platinum EBID markers to aid in gauging thinning progression. A thicker coating of ion beam-induced deposition (IBID) tungsten was deposited atop the carbon and platinum.

The coated region was isolated from its surrounding via high current FIB milling and was then extracted by a micro-manipulator and attached to a copper TEM half-grid with IBID platinum. The sample was thinned with gradually lowering ion beam currents and voltages, frequently alternating which side of the lamella was being thinned, until the final sample was electron transparent. The A-TiO_2 sample was prepared in a Helios 5 UC (Thermo Fisher Scientific) equipped with a Ga^+ liquid metal ion source. A protective layer of carbon EBID was deposited followed by platinum EBID, with a final, thicker coating of tungsten IBID placed above the EBID layers. High current FIB milling was used to isolate the coated region from its surroundings before extracting it with a micro-manipulator. The lamella was welded onto a copper TEM half-grid with tungsten IBID then was thinned with progressively lower beam currents until electron transparent.

STEM analysis was performed in a probe- and image-corrected Spectra Ultra (Thermo Fisher Scientific) equipped with an X-FEG/UltiMono source operating at 300 kV accelerating voltage. Images were acquired using a 28 mrad convergence semi-angle with $\sim$ 110–130 pA beam current. Acceptance angles for the ABF, LAADF, MAADF, and HAADF (annular bright field, low-angle, medium-angle, and high-angle annular dark field, respectively) detectors were 0–11, 12–23, 23–44, and 49–200 mrad, respectively. High-resolution transmission electron microscopy (HRTEM) images were acquired using parallel illumination on a Ceta-S detector.

Electron energy loss spectroscopy (EELS) data was acquired in the STEM with a ContinuumK3 (Gatan) using the spectrometer's secondary detector (fiber-optically coupled scintillator, model 1069.EXUP). Spectrum images were acquired in DualEELS mode using a 53 mrad collection semi-angle and 0.3 eV/channel dispersion. 850 eV was applied to the drift tube to acquire EELS spectra starting at $\sim$250 and $\sim$1100 eV with exposure times of 10 and 200 ms, respectively. The decaying background was fit to a power law to extract composition information.

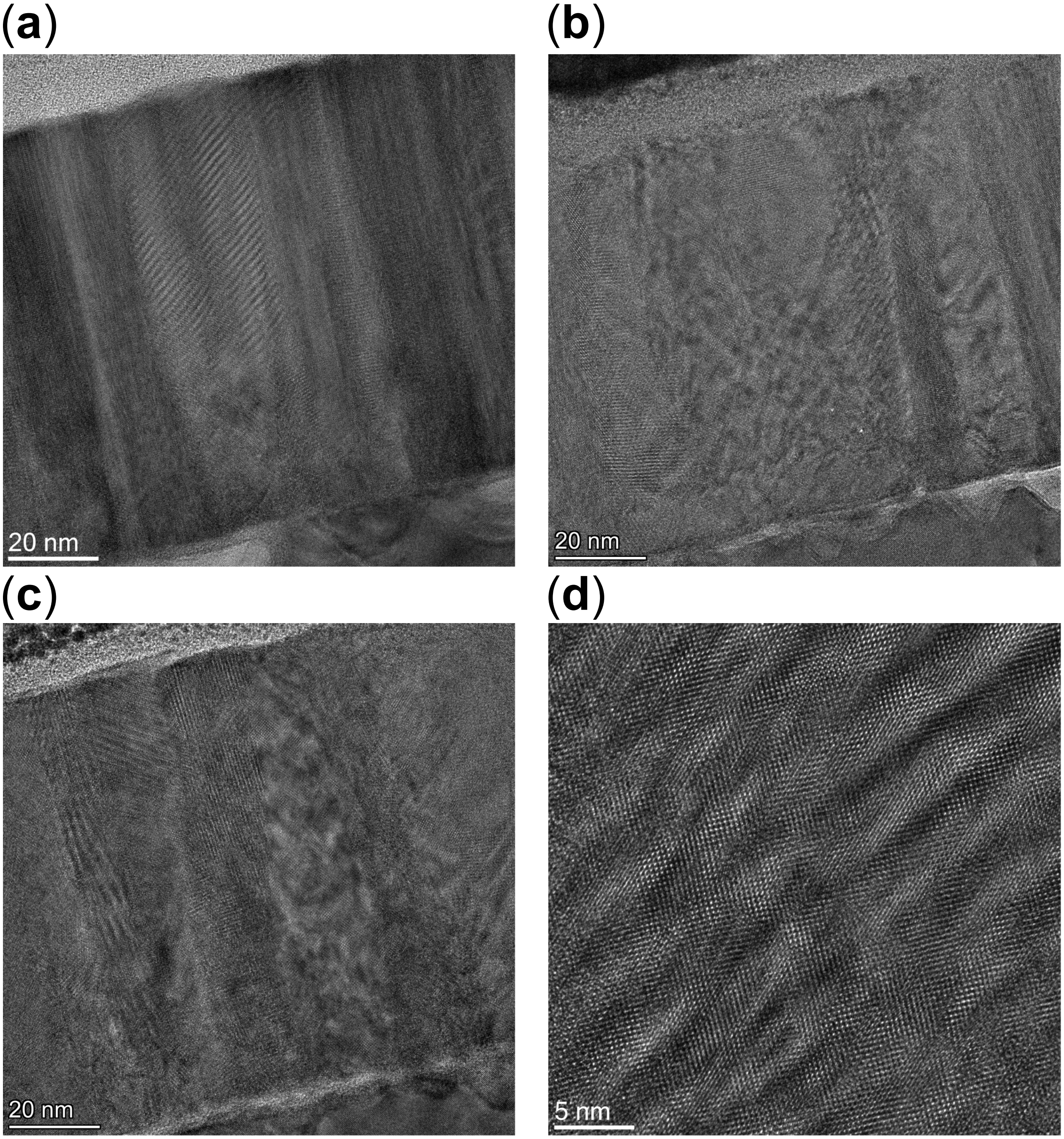

Figure S5: HRTEM images showing the presence of Moiré fringes due to crystal domain twisting/rotations in the R-TiO_2 film. (a)-(c) Images of the full film thickness showing Moiré fringes (separate regions on the film from that in Fig. 8(d) of the main text). (d) Zoom-in on Fig. 8(d) from the main text, showing the atomic-level resolution of the Moiré fringe pattern.

S2.8 Additional TEM Images

In this appendix, we provide further TEM analysis of each sample, starting with GaAs-HT-5, which features an R-TiO_2 thin film. HRTEM analysis reveals strong Moiré fringing (Fig. S5a-S5c), indicative of rotated crystals overlapping in projection. Columnar regions of these fringes appear to span through a majority of the film (Fig. S5a), consistent with the wide coverage observed in Fig. 8d of the main text. Higher-magnification analysis of the region from Fig. 8d (Fig. S5d) demonstrates atomic-level resolution amongst the fringing pattern. These results are consistent with nanocrystalline domains (see Section S2.3), allowing for multiple overlapping regions of different orientations to be captured within the sub-100 nm thick lamella.

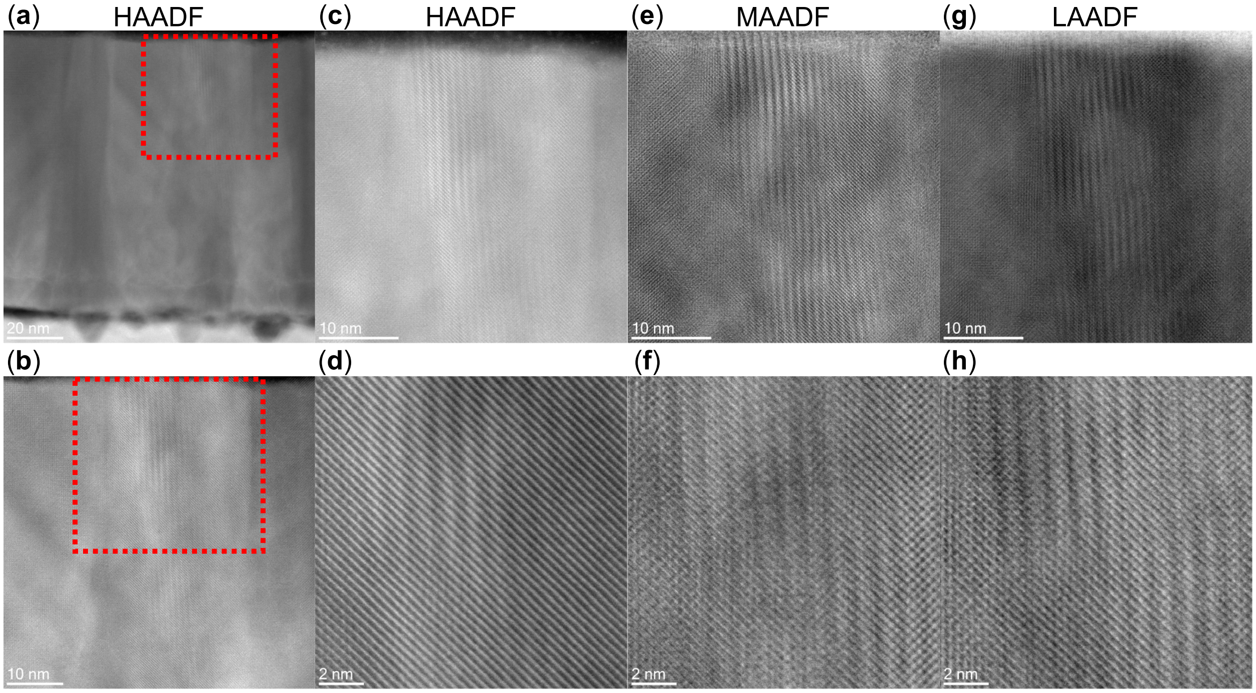

Figure S6: Further Moiré fringe STEM images of the R-TiO_2 thin film. Images (a) and (b) are HAADF-STEM scans where the zoomed-in STEM images of the dashed red box in (c),(e),(g) and (d),(f),(h) are derived, respectively. HAADF-, MAADF-, and LAADF-STEM images of (a) and (b) are included in (c) and (d), (e) and (f), and (g) and (h), respectively.

This is further exemplified within STEM imaging of the TiO_2 layer (Fig. S6), where overlapping crystal regions can also be resolved. At lower magnifications, these regions

are not obvious (Fig. S6a, S6b), but upon zooming in, they become much more apparent (Fig. S6c-h). High magnification images demonstrate how the HAADF (Fig. S6d) detects the fringing relative to the MAADF and LAADF (Figs. S6f and S6h, respectively), which are more sensitive to coherent and thermal diffuse scattering.

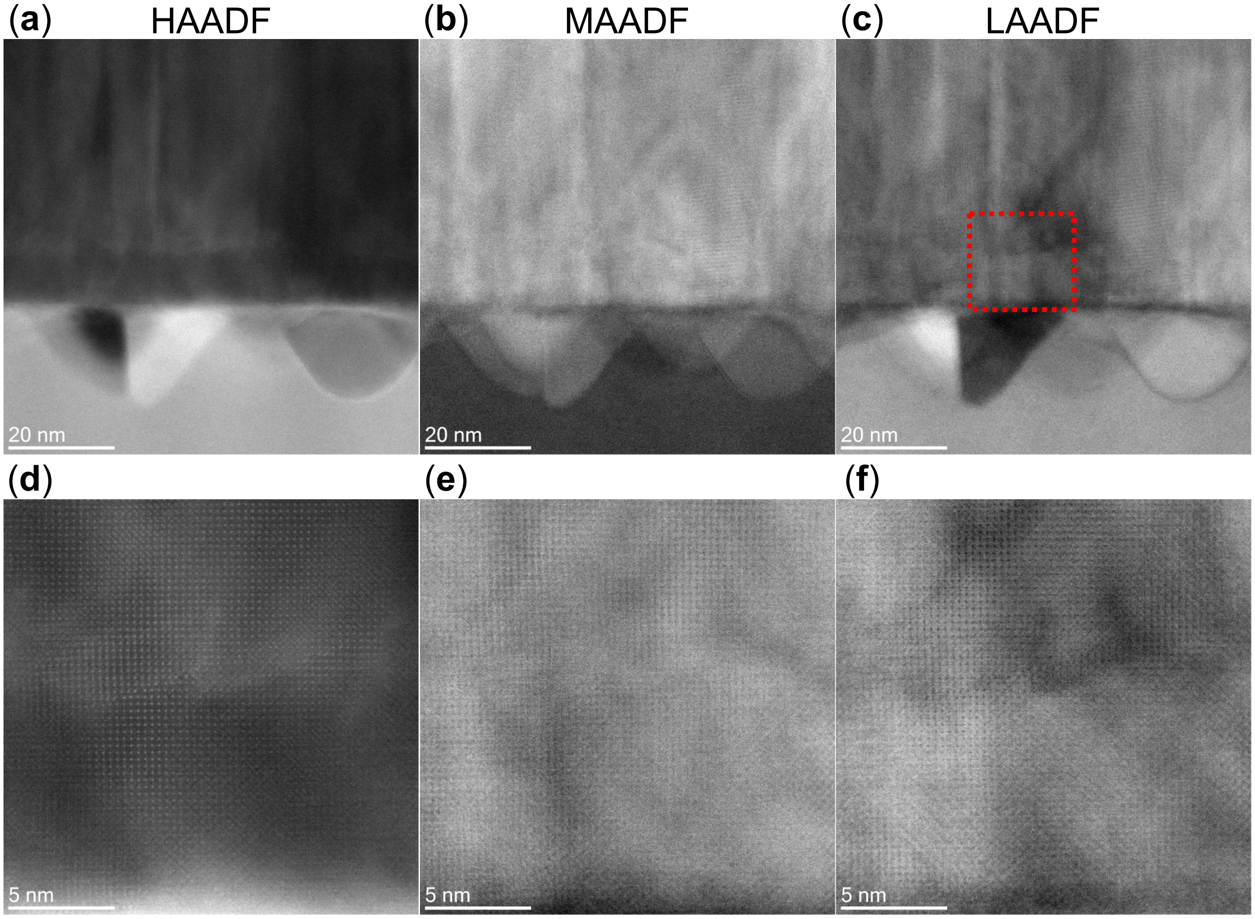

Figure S7: HAADF-, MAADF-, and LAADF-STEM images of the GaAs/TiO_x/TiO_2 interface are shown in (a), (b), and (c), respectively. Zoom-in HAADF-, MAADF-, and LAADF-STEM images of (a), (b), and (c) are included in (d), (e), and (f), respectively (R-TiO_2 thin film). Each of the images in (d)-(f) correspond to the same region in (a)-(c) demarcated by the dashed red box in (c). Zoom-in image (d) is used in Fig. 9(b) of the main text.

There appears to be a distinct TiO_x/TiO_2 interface across the sample, clearly imaged with the HAADF detector (Fig. S7a) as a bright line between the two oxides. MAADF imaging (Fig. S7b) does not detect this interface as readily, whereas LAADF (Fig. S7c) imaging views it as a dark line. Multiple defect sites are observed at the TiO_x/TiO_2 boundary (Fig. S7d-f).

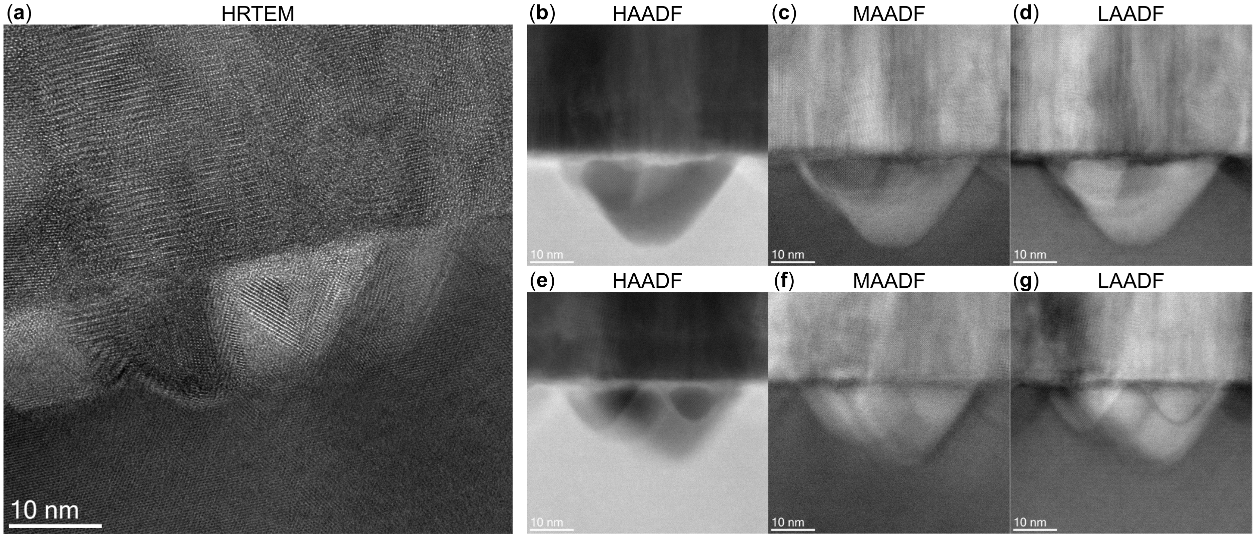

Figure S8: (a) HRTEM image showing the atomic-level resolution of a Ga deficient pit commonly seen in the combined TiO_x-GaAs interface (R-TiO_2 thin film). HAADF-, MAADF- and LAADF-STEM images of two separate regions containing multiple Ga deficient pits are shown in (b) and (e), (c) and (f), and (d) and (g), respectively.

Additional imaging of the Ga-deficient pits (Fig. S8) demonstrates the polycrystalline nature of these pits along with the nucleation of defects near these sites, as demonstrated within Fig. 8 and 9 of the main text. Regions of textured Ga-deficient pits appear to correlate to likewise textured TiO_x/TiO_2 (Fig. S8a). HAADF-STEM imaging is beneficial for determining crystal orientation (Fig. S7a, S8b, S8e) near regions of high-density pits while simultaneously providing atomic contrast. Co-acquired MAADF (Fig. S7b, S8c, S8f) clears some of the atomic contrast and allows for clarification of diffraction contrast, while LAADF imaging (Fig. S7c, S8d, S8g) further accentuates diffraction differences between the pits.

To complement the R-TiO_2 microstructural analysis, we present additional TEM characterization of the A-TiO_2 thin film grown on arsenic-capped GaAs (sample GaAs-LT-7). Figure S9(a)-(d) shows HRTEM images revealing the crystalline quality and defect structure of the anatase film. Moiré patterns are again observed, suggesting that the low-temperature growth kinetics and improved substrate surface quality do not suppress the formation of rotational variants.

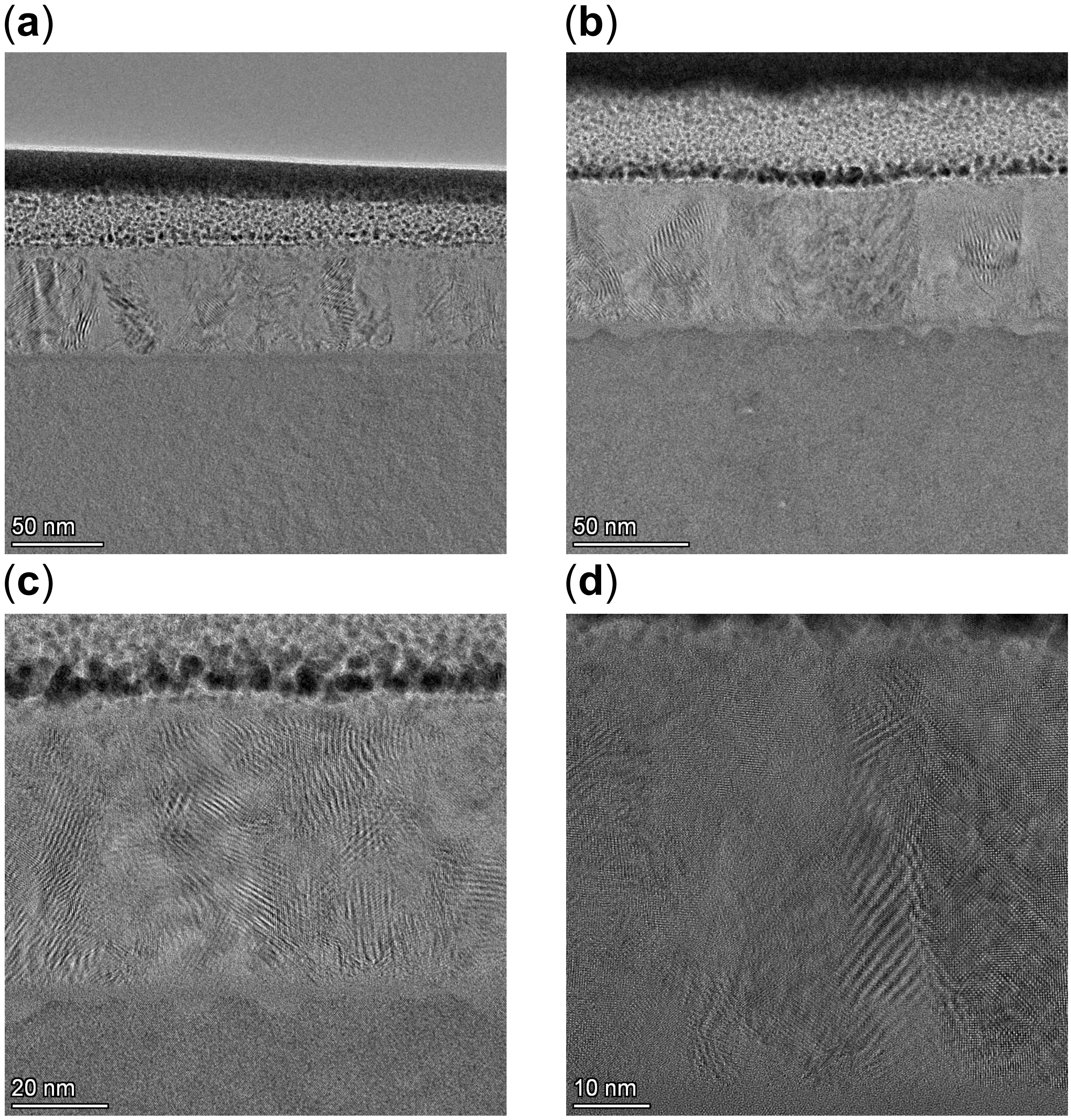

Figure S9: HRTEM images revealing the presence of Moiré fringing in the A-TiO_2 film are shown in (a)-(d) with increasing magnification. Note that in each of the images shown in (a)-(d), different lamella cross-section locations are utilized.

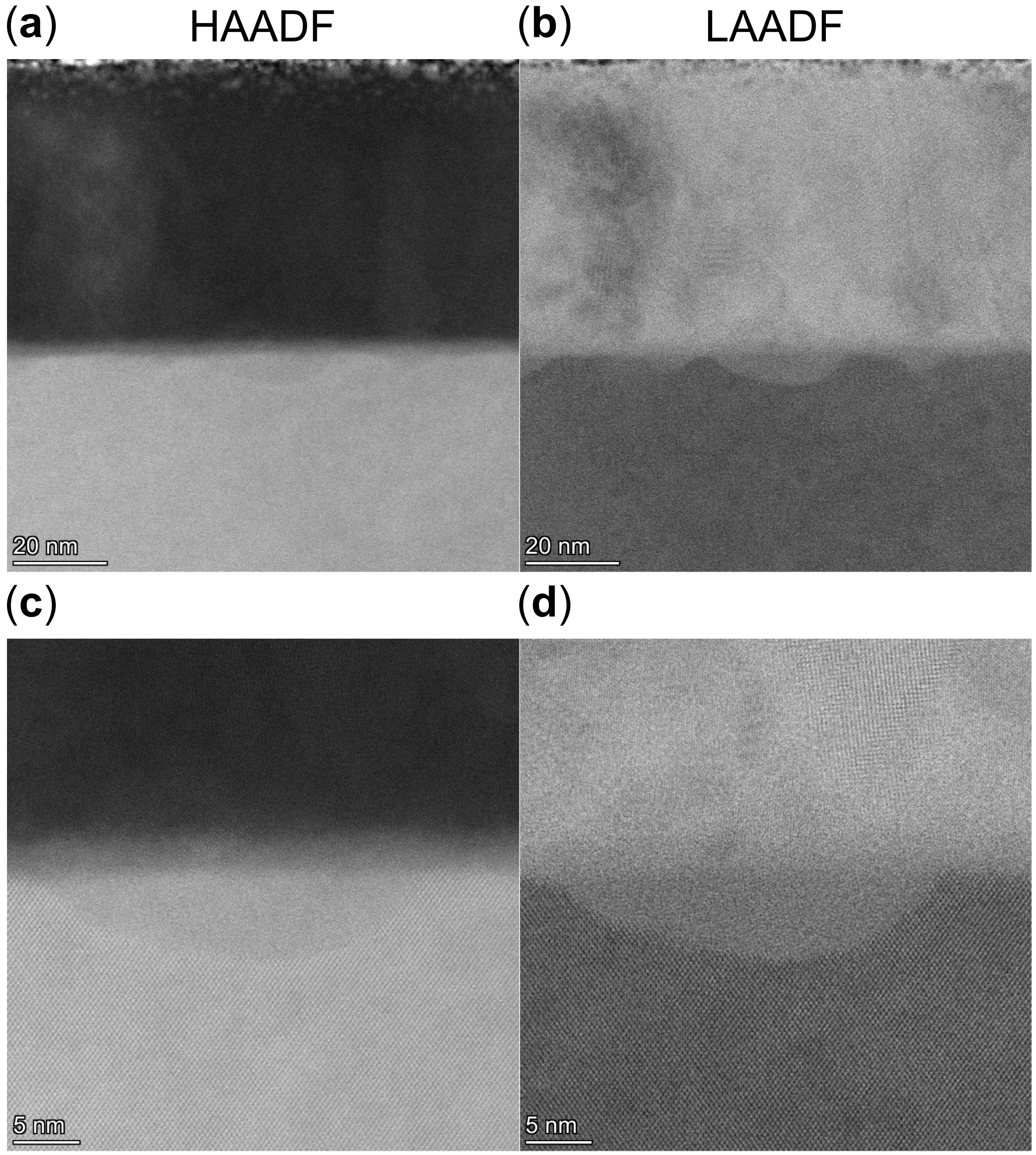

Figure S10: HAADF-STEM and LAADF-STEM images of the gallium-deficient pits present in the GaAs substrate for the A-TiO_2 film are shown in (a) and (b), respectively. Higher magnification images of the same pits shown in (a) and (b) are shown in (c) and (d), respectively.

The GaAs/TiO_x/TiO_2 interface in the A-TiO_2 sample (Figure S10) presents a stark contrast to the oxide-desorbed case. The arsenic-capped GaAs surface shows minimal pit formation that characterized the high-temperature oxide desorption process. Although some interfacial roughness remains, the characteristic gallium-deficient voids observed in Figure S8 are minimal and much shallower when present. This preservation of surface integrity is attributed to two factors: (1) the protective arsenic cap prevents native oxide formation during sample transfer, eliminating the need for aggressive thermal desorption, and (2) the lower growth temperature (390 °C vs. $>$ 540 °C) reduces the driving force for Ga out-diffusion and surface reconstruction. HAADF- and LAADF-STEM imaging (Figures S10(a),(c) and S10(b),(d), respectively) at the TiO_x/TiO_2 interface reveals a more gradual transition compared to the R-TiO_2 case, with the amorphized oxygen-deficient buffer layer thickness ($\approx$ 1.4 nm) being significantly thinner than in the high-temperature growths ($\approx$ 10 nm). The thinner (amorphous) buffer may also contribute to the phase selectivity, where anatase formation could be favored at lower buffer thicknesses.

S3. Interface and Defect Analysis

S3.1 Estimating the Diffusion Constant of Gallium in TiO_2

The procedure used to estimate the Ga diffusion constant from STEM/EELS profiles is described below. During the oxygen-pressure ramp when the growth is paused, Ga atoms accumulate near the TiO_x/TiO_2 interface. To quantify the diffusion behavior, we fit the Ga EELS intensity in each region separately to an exponential decay function $C(z) = A\exp[-(z - z_0)/\lambda] + c$, where λ is the characteristic decay length and A, z_0, and c are fitting parameters. The resulting fits and parameters are shown in Fig. S11. The diffusion constant was calculated from the fitted decay length as λ using $D_{\mathrm{Ga}}(t) = \lambda^2/4t$, where t is the effective diffusion time at a given substrate temperature T. The associated uncertainty was determined by propagation of the covariance matrix element corresponding to σ_λ as $\sigma_D = \lambda\sigma_\lambda/2t$.

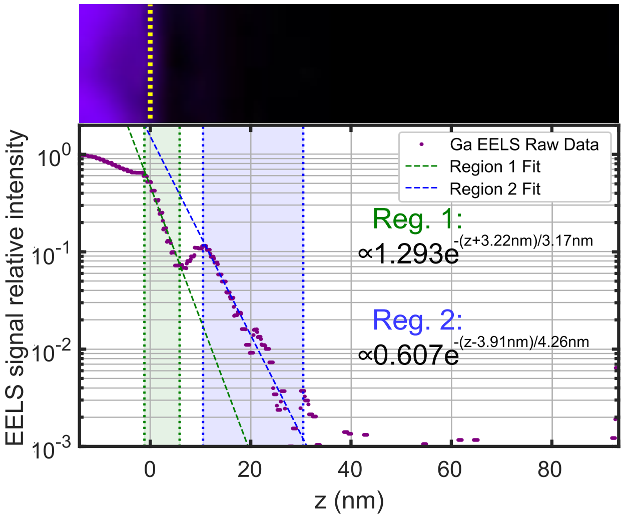

Figure S11: Integrated gallium EELS signal as a function of position from the TiO_2/GaAs interface. The resulting negative exponential fits utilized to extract the gallium diffusion constant are included in the plot.

For sample GaAs-HT-5 on which the EELS measurement was carried out, the substrate temperature was ramped linearly from 545 °C to 495 °C over the 20 min growth time

of the TiO_2 thin film. Additionally, it took around 30 min to cool the sample to room temperature after the growth. Because the precise temperature profile during cooldown is not well constrained, we evaluate D_{Ga} at two representative diffusing times, t = 45 min and t = 60 min, to bound the plausible range of D_{Ga}. This approach implicitly captures both the time dependence of the diffusion process and its temperature dependence across the 495 − 545 °C range. The resulting values are summarized in Table S13.

Table S13: Extracted Ga diffusion constants from the negative exponential fits.

**Oxide host**	λ [nm]	$\mathbf{D}_{Ga}$**(t = 45 min)** [$\mathrm{cm^2\,s^{-1}}$]	$\mathbf{D}_{Ga}$**(t = 60 min)** [$\mathrm{cm^2\,s^{-1}}$]
TiO_x (Region 1)	3.2(3)	$9.3(2)\times10^{-18}$	$7.0(1)\times10^{-18}$
TiO_2 (Region 2)	4.3(1)	$1.68(7)\times10^{-17}$	$1.26(5)\times10^{-17}$

For context, Table S14 lists the reported diffusion constant of Ga in SiO_2 at comparable temperatures. The extracted D_{Ga} values for TiO_2 are of the same magnitude, suggesting that Ga diffusion proceeds at rates similar to those in other dense oxide matrices.

Table S14: Literature Ga diffusion constants in SiO_2.

$\mathbf{D}_{Ga}$**(T)** [$\mathrm{cm^2\,s^{-1}}$]	$\mathbf{D}_{Ga}$**(T=495 °C)** [$\mathrm{cm^2\,s^{-1}}$]	$\mathbf{D}_{Ga}$**(T=545 °C)** [$\mathrm{cm^2\,s^{-1}}$]
$2.44\exp(-2.57\ \mathrm{eV}/k_BT)$ †	3.3×10^{-17}	3.6×10^{-16}
$0.73\exp(-2.46\ \mathrm{eV}/k_BT)$ ‡	5.2×10^{-17}	5.1×10^{-16}

†Reported in Ref.[9]
‡Reported in Ref.[10]

S3.2 EELS Relative Ti/O Atomic Composition

Figure S12 presents the relative EELS signal contributions from titanium and oxygen across the R-TiO_2 thin film grown on GaAs. At each position along the growth direction, the lateral (column-wise, parallel to the interface) EELS intensities were integrated and normalized by the total elemental signal (Ti, O, Ga, and As) at that distance z. The EELS colormaps were formatted so that a perfectly stoichiometric film (i.e., TiO_2) would correspond to Ti and O fractions of 0.5.

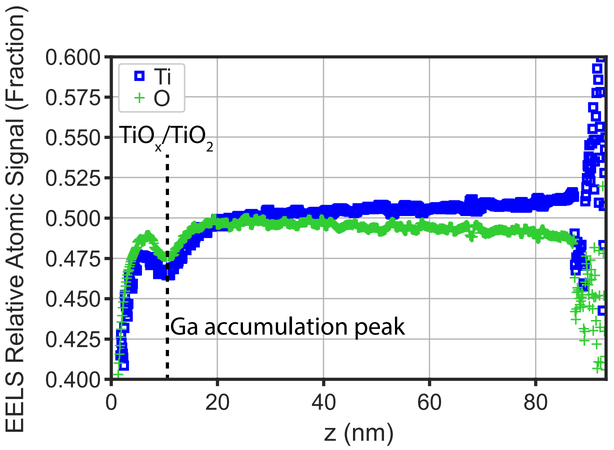

Figure S12: Relative EELS signal from titanium (Ti) and oxygen (O) of the rutile TiO_2 thin film grown on GaAs (Sample name: NS21).

Both Ti and O signals exhibit subtle variations with distance from the interface. Within the first $\sim$ 20 nm from the GaAs interface, the relative O and Ti contributions modulate due to Ga diffusion and accumulation near the TiO_x/TiO_2 interface. At the Ga accumulation region, Ti and O signals decrease by approximately 2.5%, and the Ti signal remains consistently lower than that of O, consistent with Ga diffusion via and incorporation at Ti sites. Stoichiometric TiO_2 is recovered by $z = 20$ nm, where the Ga EELS signal (Fig. S11) falls below 1% of the bulk GaAs level.

Beyond $z = 20$ nm, the relative O signal gradually decreases with distance, suggesting the accumulation of oxygen vacancies during film growth. Although deposition occurred under oxygen-rich conditions (20 mTorr), the relatively low substrate temperature and moderate growth rate (0.85 $\text{Å}\,\text{s}^{-1}$; 5 Hz laser repetition with 0.17 Å per shot) likely limited complete suppression of oxygen vacancies.[1] Such depth-dependent deviations from stoichiometry may influence defect chemistry and dopant incorporation in similar oxide/semiconductor heterostructures. Future investigations of Er^{3+}:TiO_2 films on III-V substrates would benefit from high-resolution compositional mapping (e.g., atom probe tomography) to more precisely resolve Ti, O, and Er distributions as a function of distance from the interface.

S3.3 Rate Equation Model of Depth-resolved Defects

A simple rate equation was employed to qualitatively model oxygen vacancy (V_O) formation and diffusion within the TiO_2 buffer layer before, during, and after oxygen ramping. The objective of these calculations is to elucidate how (i) buffer layer growth temperature and (ii) the number of laser shots (i.e., buffer layer thickness) collectively influence the resulting TiO_2 phase due to vacancy accumulation and strain.

The partial differential equation governing the system is based on Fick's second law of diffusion:[11]

$$\frac{\partial n_v}{\partial t} = D\frac{\partial^2 n_v}{\partial z^2} + R_{\text{inc}}(t), \tag{6}$$

where $n_v(z,t)$ is the oxygen vacancy concentration (atoms/cm^3), D is the bulk diffusion coefficient of oxygen (cm^2/s), $R_{\text{inc}}(t)$ is the oxygen vacancy incorporation rate (atoms/cm^3/s), and z is the distance from the GaAs surface (cm). A growth rate of 0.017 nm per laser shot and a 5 Hz laser repetition rate yield an effective deposition rate of $R_{\text{growth}} = 0.085\ \text{nm}\,\text{s}^{-1}$. The simulation grid is spatially discretized with a resolution $\Delta z = 0.004$ nm, giving $N_z = L/\Delta z$ grid points, where $L = N_s R_{\text{growth}}$ is the total film thickness for given number of laser shots N_s. The growth and total times are defined as

$$t_{\text{growth}} = L/R_{\text{growth}}, \tag{7}$$

$$t_{\text{total}} = t_{\text{growth}} + t_{\text{purge}}, \tag{8}$$

where the oxygen purge time t_{purge} corresponds to the ramp time required for the chamber O_2 pressure to reach 20 mTorr, which was varied between 60 s and 300 s.

The physical parameters used in the simulation were selected from prior studies on oxides,[12] with A-TiO_2 values[13] chosen where available. These include characteristic values for oxygen-vacancy migration and surface kinetics, summarized in Table S15. The corresponding Arrhenius equations for thermally-activated bulk diffusion coefficient (D) and the surface

reaction rate constant (k_s) are:

$$D = D_0 \exp\left(-\frac{E_m}{k_B T}\right), \tag{9}$$

$$k_s = k_{s0} \exp\left(-\frac{E_s}{k_B T}\right). \tag{10}$$

Table S15: Physical parameters used in the rate-equation model for oxygen-vacancy diffusion in TiO_2.

**Parameter Description**	**Symbol / Value**
Vacancy migration attempt frequency	$\nu_0 = 10^{13}\ \mathrm{s}^{-1}$
Bulk diffusion rate at 0 K	$D_0 = 10^{-6}\ \mathrm{cm}^2\,\mathrm{s}^{-1}$
Surface diffusion rate at 0 K	$k_{s0} = 0.4\ \mathrm{cm}\,\mathrm{s}^{-1}$
Activation energy for bulk diffusion	$E_m = 1.0$ eV
Activation energy for surface diffusion	$E_s = 0.7$ eV
Density of oxygen sites in the lattice	$N_{\mathrm{sites}} = 10^{22}\ \mathrm{cm}^{-3}$
Growth temperature	$T = 350$ °C

The time-dependent oxygen pressure profile is modeled as:

$$p_{O_2}(t) = \begin{cases} p_{O_2}^{\text{initial}} & \text{if } t < t_{\text{growth}} \\ p_{O_2}^{\text{initial}} + (p_{O_2}^{\text{final}} - p_{O_2}^{\text{initial}})\frac{t - t_{\text{growth}}}{t_{\text{purge}}} & \text{if } t \geq t_{\text{growth}} \end{cases} \tag{11}$$

During growth ($t < t_{\text{growth}}$), oxygen vacancies are incorporated at a rate of R_{inc} determined by the deposition rate $R_{\text{dep}} \equiv R_{\text{growth}} \times N_{\text{sites}}$ as

$$R_{\text{inc}} = \frac{f_v \times R_{\text{dep}}}{\Delta z} \quad \text{atoms/cm}^3\,\text{s}^{-1}, \tag{12}$$

where $f_v = 0.01$ is the fractional V_{O} concentration prior to purging the chamber with O_2.

The second spatial derivative of V_{O} density is approximated using central differences as[11]

$$\left.\frac{\partial^2 n_v}{\partial z^2}\right|_i \approx \frac{n_{v,i+1} - 2n_{v,i} + n_{v,i-1}}{(\Delta z)^2} \tag{13}$$

for interior grid points ($i = 1, 2, \ldots, N_z - 2$). At the interface ($z = 0$), the boundary condition

balances diffusive flux and surface reaction kinetics:[12]

$$-D\frac{\partial n_v}{\partial z}\bigg|_{z=0} = k_s(n_v|_{z=0} - n_{v,eq}). \tag{14}$$

Using forward differences, the first and second derivatives of n_v become:

$$\frac{\partial n_v}{\partial z}\bigg|_{z=0} \approx \frac{n_{v,1} - n_{v,0}}{\Delta z}, \tag{15}$$

$$\frac{\partial^2 n_v}{\partial z^2}\bigg|_{z=0} = \frac{2(n_{v,1} - n_{v,0})}{(\Delta z)^2} - \frac{2k_s(n_{v,0} - n_{v,eq})}{D\Delta z}. \tag{16}$$

A no-flux boundary condition is imposed at the back surface ($z = L$):

$$\frac{\partial n_v}{\partial z}\bigg|_{z=L} = 0. \tag{17}$$

The resulting system of ordinary differential equations is:

$$\frac{dn_{v,i}}{dt} = D\frac{\partial^2 n_v}{\partial z^2}\bigg|_i + R_{\text{inc},i}(t) \tag{18}$$

for $i = 0, 1, 2, \ldots, N_z - 1$. The system is solved using the `solve_ivp` function of `SciPy`, which employs the Radau method.[14] The Radau method is well-suited for stiff systems, such as advection-diffusion-reaction type systems[15] similar to our scenario. Here, the oxygen vacancy diffusion creates a sharp concentration gradient near the surface ($z = 0$) and includes coupled fast surface kinetics (k_s) and slower bulk diffusion (D). We specify an initial condition corresponding to zero vacancies (i.e., no pre-existing TiO_2 film). Relative and absolute tolerances of 10^{-8} (10^{-13}) and 10^{-10} (10^{-15}), respectively, are used for simulations with t_{purge} of 60 s (300 s).

The average vacancy density within buffer layers of varying thickness grown in vacuum and for two different purge times (the interval during which O_2 pressure was ramped) is shown in Fig. S13. As expected, films grown without oxygen exposure (blue circles) retain

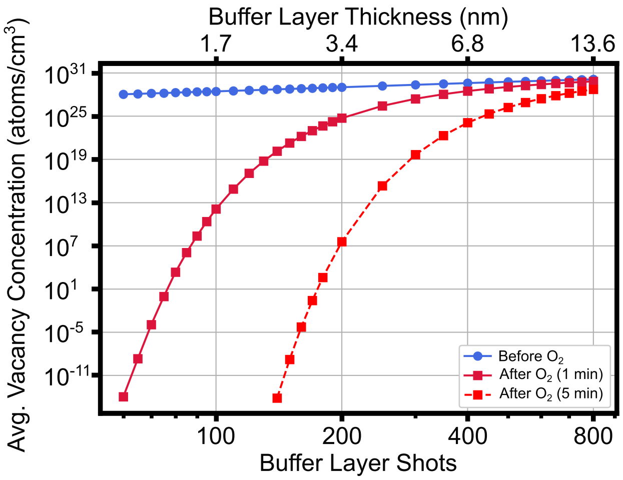

Figure S13: Average V_O density, calculated by taking the mean of the depth-resolved V_O density, for various buffer layer shots before and after O_2 purge.

a high and nearly constant vacancy concentration across all thicknesses, reflecting vacancy incorporation during TiO_2 deposition under vacuum conditions. Upon O_2 introduction, the vacancy concentration decreases sharply for thin buffer layers and continues to drop with extended purge duration (red symbols), indicating active annihilation of oxygen vacancies. Thinner buffer layers exhibit a more complete reduction in V_O density, consistent with the shorter diffusion path length and more effective surface reoxidation. In contrast, thicker buffers (>10 nm) retain a substantial vacancy population even after prolonged oxygen exposure, implying diffusion-limited reoxidation kinetics.

The results for the specific cases of 200 and 600 buffer layer shots, corresponding to ~3.5 and ~10 nm thicknesses, are shown in Fig. S14(a) and S14(b), respectively. Here, 0 depth denotes the TiO_2–air interface, where oxygen is introduced and reacts with surface vacancies before diffusing inward. Before O_2 exposure (blue curves), both films exhibit a nearly uniform V_O concentration throughout the thickness, reflecting the vacancy incorporation that occurs under vacuum growth conditions. After O_2 ramping, the near-surface vacancy concentration decreases significantly, resulting in a sharp gradient that varies exponentially with depth as oxygen diffuses into the film. This gradient becomes more pronounced and extends deeper for longer purge durations (60 s vs. 300 s, red curves), consistent with time-dependent

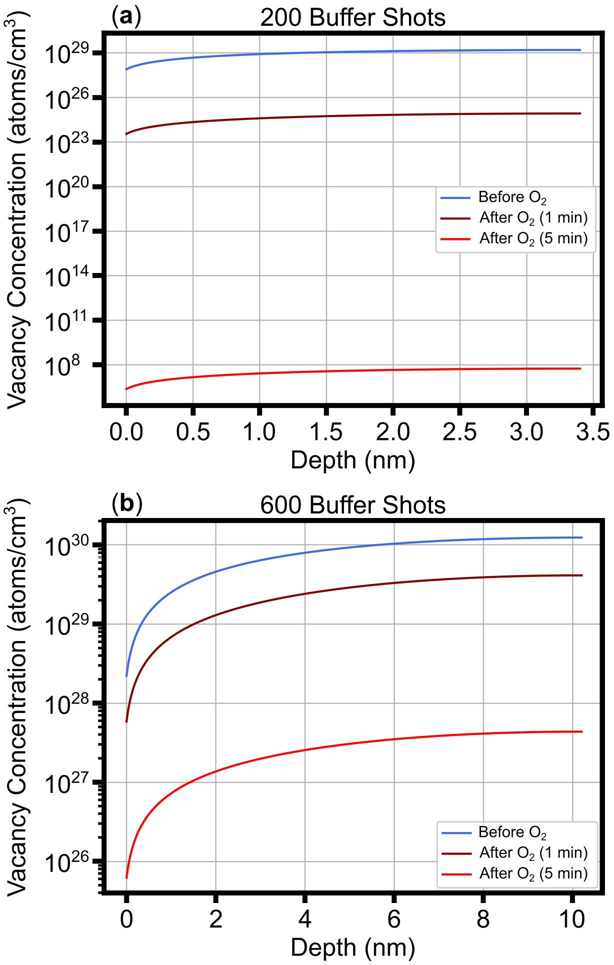

Figure S14: Depth-dependent oxygen vacancy distribution for (a) 200 and (b) 600 buffer layer shots before and after O_2 purge.

diffusion-limited oxidation. In the thinner (200-shot) buffer, oxygen can fully penetrate the film thickness within the purge times, leading to near-perfect stoichiometric recovery. In contrast, the thicker (600-shot) buffer retains a substantial vacancy concentration in the interior even after 300 s of oxygen exposure, indicating that the reoxidation front is kinetically limited by the relatively low bulk diffusivity of oxygen at a growth temperature of 350 °C. Overall, the depth-dependent profiles confirm that vacancy annihilation proceeds from the surface inward and that the efficiency of oxygen incorporation strongly depends on both film thickness and purge duration.

S4. Optical and Spectroscopic Characterization

S4.1 Optical Raman Spectroscopy Data

Table S16: Raman spectroscopy peak locations for A-TiO_2 thin films showing the B_{1g} ($\sim$ 399 cm^{-1}), B_{1g}/A_{1g} ($\sim$ 513/519 cm^{-1}) and E_g ($\sim$ 639 cm^{-1}) characteristic peaks.

**Sample**	**Substrate**	**Er-Doped?**	$\mathbf{B_{1g}}$ (cm^{-1})	$\mathbf{B_{1g}/A_{1g}}$ (cm^{-1})	$\mathbf{E_g}$ (cm^{-1})
GaAs-LT-4	GaAs	Yes	399	510	645
GaAs-LT-1	GaAs	Yes	396	520	636
GaAs-LT-5	GaAs	Yes	404	512	634
GaAs-LT-2	GaAs	Yes	399	523	647
GaAs-LT-3	GaAs	Yes	399	517	652
GaAs-LT-7	GaAs	Yes	399	520	644
GaAs-LT-8	GaAs	Yes	402	512	642
GaAs-LT-9	GaAs	Yes	402	520	639
GaAs-LT-10	GaAs	Yes	399	510	642
GaAs-LT-11	GaAs	Yes	399	510	637
GaAs-LT-12	GaAs	Yes	399	517	642
GaAs-LT-13	GaAs	Yes	397	517	650
GaSb-LT-1	GaSb	Yes	397	507	634

Optical Raman spectroscopy was employed to identify the TiO_2 polymorphs of films synthesized on GaAs and GaSb substrates, particularly when XRD measurements were inconclusive due to low diffraction efficiencies. For completeness, Table S16 lists the peak positions for A-TiO_2 films, including the B_{1g} ($\sim$ 399 cm^{-1}), B_{1g}/A_{1g} ($\sim$ 513/519 cm^{-1}) and E_g ($\sim$ 639 cm^{-1}) modes. Similarly, Table S17 summarizes the characteristic Raman peak positions of R-TiO_2, corresponding to E_g ($\sim$ 449 cm^{-1}) and A_{1g} ($\sim$ 614 cm^{-1}) modes. Although not tabulated, TiO_2 films grown on Si substrates were confirmed to be exclusively anatase phase.

Additionally, Raman spectra of all substrates used in the study are presented in Fig. S15. These measurements, obtained from 10 mm $\times$ 10 mm substrate pieces, serve as calibration references to identify and distinguish substrate-related peaks from film contributions. The characteristic features of GaAs (267 and 291 cm^{-1}), GaSb (234 and 236 cm^{-1}), Si (520 cm^{-1}), and R-TiO_2 (449 and 614 cm^{-1}) are all clearly resolved.

Table S17: Raman spectroscopy peak locations for R-TiO_2 thin films showing the E_g ($\sim$ 449 cm^{-1}) and A_{1g} ($\sim$ 614 cm^{-1}) characteristic peaks.

**Sample**	**Substrate**	**Er-Doped?**	$\mathbf{E}_g$ (cm^{-1})	$\mathbf{A}_{1g}$ (cm^{-1})
GaAs-HT-2	GaAs	No	445	612
GaAs-HT-4	GaAs	Yes	451	608
GaAs-HT-7	GaAs	No	444	614
GaAs-LT-6	GaAs	Yes	447	–
GaAs-HT-3	GaAs	Yes	454	605
GaSb-HT-1	~~GaAs~~GaSb	Yes	441	598
GaSb-HT-2	GaSb	No	441	606
GaSb-HT-3	GaSb	No	439	608

Figure S16 shows the Raman spectrum of an Er^{3+}:TiO_2 film grown on a rutile TiO_2 (110) substrate. The dominant E_g and A_{1g} modes characteristic of the rutile phase are evident, confirming epitaxial alignment between the doped film and the crystalline substrate. The absence of additional broad features further suggests that Er incorporation does not induce significant lattice distortion or amorphization under these growth conditions.

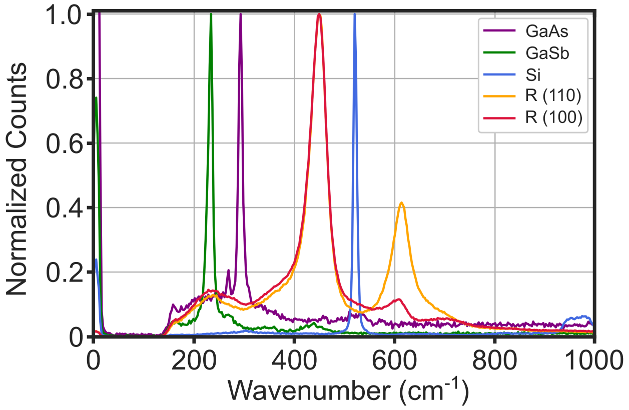

Figure S15: Raman spectra of the bare substrates, which were employed to delineate the Raman modes of the thin films.

Figure S17 compares Raman spectra of Er^{3+}:TiO_2 films grown on SOI(100) substrates under different growth conditions. In Fig. S17(a), films grown with TiO_2 buffer layers at lower temperature (365 celsius) display sharper anatase-like features relative to those grown at 500 celsius, where increased intensity in the 400–500 cm^{-1} region indicates improved crystallinity. In contrast, substituting the TiO_2 buffer with CeO_2 under identical thermal

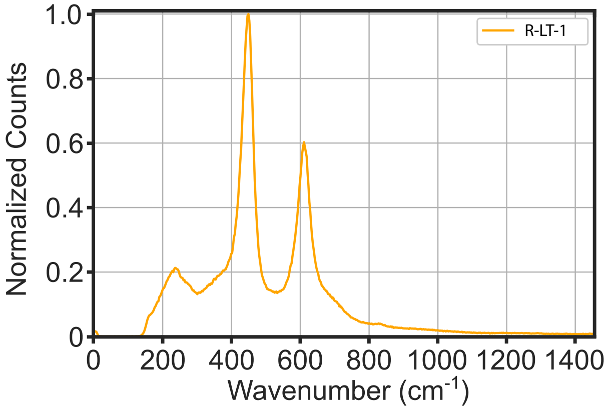

Figure S16: Optical Raman Spectroscopy results for Er^{3+}:TiO_2 thin film synthesized on a R-TiO_2 (110) substrate.

conditions (sample Si-HT-2) slightly suppresses the high-frequency modes, suggesting minor strain relaxation or altered nucleation dynamics at the interface. Figure S17(b) further highlights that increasing the CeO_2 buffer thickness (from 70 to 200 shots) enhances overall peak definition, consistent with the formation of a more ordered TiO_2 lattice. Future studies could further explore the role of the CeO_2 buffer in determining the TiO_2 phase and thin-film quality grown on Si substrates.

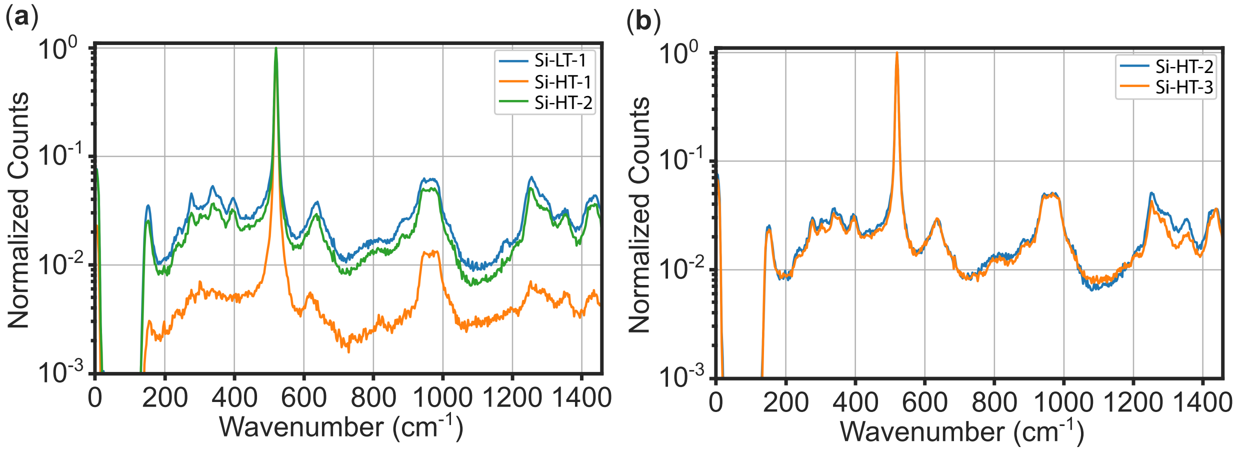

Figure S17: Optical Raman Spectroscopy results for Er^{3+}:TiO_2 thin films synthesized on (100) SOI substrates. (a) Differences in Raman spectra as a function of growth temperature and buffer material are shown for samples Si-LT-1 (TiO_2 buffer; $T_{grow} = 365\ ^{\circ}C$), Si-HT-1 ($TiO_2$ buffer; $T_{grow} = 500\ ^{\circ}C$), and Si-HT-2 ($CeO_2$ buffer; $T_{grow} = 500\ ^{\circ}C$). (b) Differences in Raman spectra as a function of ceria buffer shots for samples Si-HT-2 (70 buffer shots) and Si-HT-3 (200 buffer shots) grown at 500 $^{\circ}C$.

S4.2 Optical Setup for PLE Spectroscopy

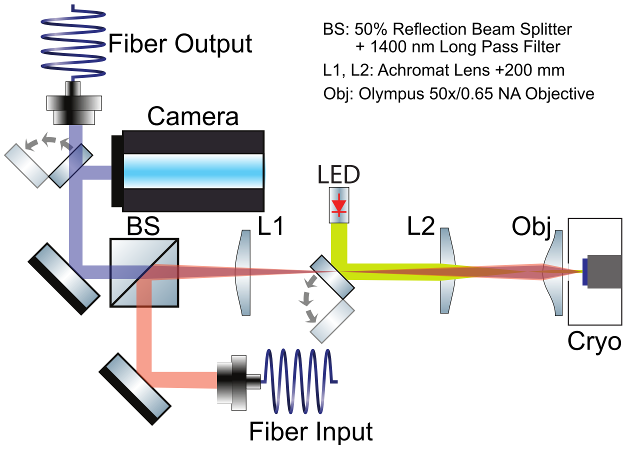

Figure S18: Schematic of the experimental setup for optical characterization of the Er^{3+}:TiO_2 samples. Laser excitation and fluorescence collection are performed through a high-NA objective (Obj). A 50/50 beamsplitter (BS) and a 1400 nm long-pass filter separate the excitation and collection paths. The LED and InGaAs camera are used for sample imaging and positioning.

Photoluminescence Excitation (PLE) spectroscopy was conducted on Er^{3+}:TiO_2 samples cooled to 5.2 K in a closed-cycle helium cryostat (Montana Instruments Cryocore). Excitation was provided by a telecom-band continuous-wave fiber-coupled laser (Pure Photonics PPCL590), tunable from 191.5 THz (1565.496 nm) to 197.5 THz (1517.936 nm). The optical setup used to excite and collect fluorescence from the sample is illustrated in Fig. S18. The fiber-coupled laser was collimated using a 20 mm-focal-length achromatic fiber collimator, producing a laser beam of 4.2 mm diameter at the "Fiber input". The collimated laser beam was relayed to the back focal plane of a high-numerical-aperture microscope objective (Olympus LCPLN50XIR; NA = 0.65) using a pair of achromatic lenses ($L1$ and $L2$), each with a focal length of 200 mm. The objective (Obj) focused the laser beam into a diffraction-limited spot ($1/e^2$-width = 1.4 µm) on the sample through a 0.5 mm-thin AR-coated vacuum window on the cryostat.

Fluorescence emitted by the sample was collected using the same objective and routed collinearly with the excitation path. A 50/50 beam splitter (BS; Thorlabs BSW29R) sepa-

rated the emission from the excitation laser beam, and the transmitted fluorescence passed through a 1400 nm long-pass filter to reject stray light. The filtered emission was then coupled into a single-mode optical fiber at the "Fiber Ouptut", serving as a spatial filter to collect light selectively from the excitation volume. For sample alignment and imaging, a near-infrared LED (Thorlabs LED1550E) illuminated the sample, and the reflected image was captured on an InGaAs camera.

To implement PLE spectroscopy, the excitation laser was intensity-modulated, and the fluorescence detection was time-gated to suppress scattered laser light. The modulation and gating were achieved using acousto-optic modulators (AOMs) synchronized by a digital delay generator (DDG), as described below and illustrated in Fig. S19.

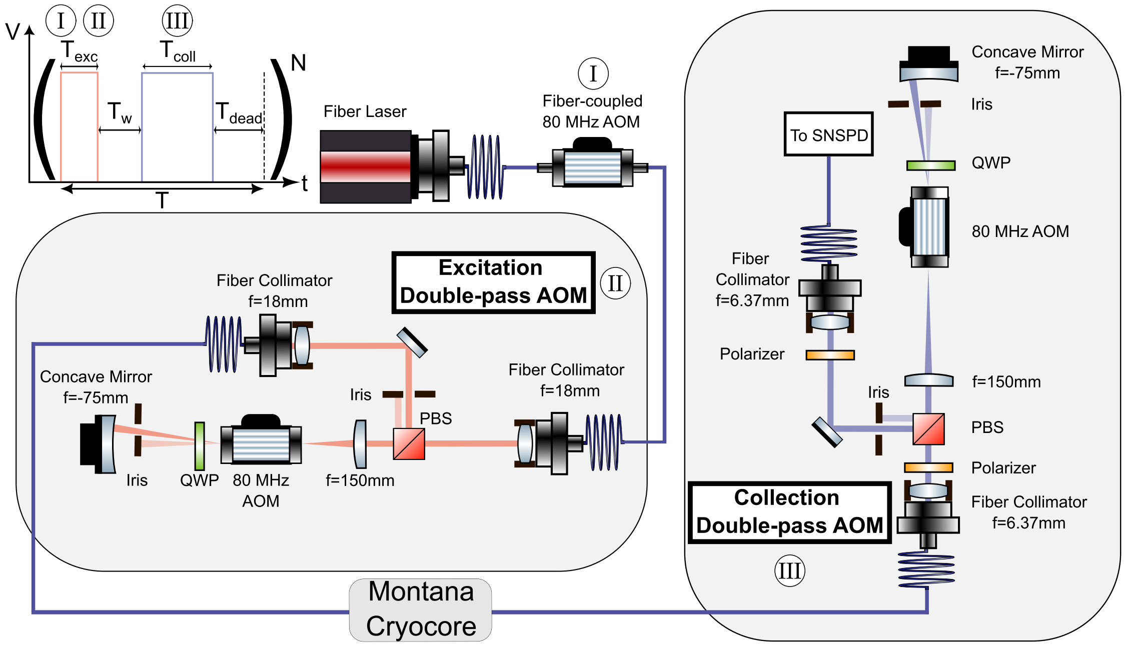

Figure S19: Schematic of the pulse-train setup used to resonantly excite Er^{3+} ions in TiO_2 and temporally gate fluorescence detection. The continuous-wave laser is modulated by a fiber-coupled AOM and a free-space double-pass AOM to generate excitation pulses of width Texc. The fluorescence is temporally filtered by a collection double-pass AOM with pulse width T_{coll} delayed by T_{w} after excitation. Excitation modulation and photodetection are electronically synchronized using a multichannel digital delay generator (not shown) to generate the relevant TTL pulses with a repetition period T. A superconducting nanowire single-photon detector (SNSPD) received the time-gated fluorescence and the measured photodetections over N pulse periods were captured.

The excitation laser intensity was modulated using a pair of 80 MHz AOMs in series–first by a fiber-coupled AOM (Agiltron AOMF-L52134332; intensity modulation contrast of $\sim$ 52 dB) and then by a free-space double-pass AOM setup (Agiltron AOMS-115001111; intensity modulation contrast of $\sim$ 61 dB). Both AOMs were synchronized via a 4-channel digital delay generator (DDG; Berkeley Nucleonics Model 588). The DDG generated a rectangular TTL pulse of width $T_{\text{exc}} = 0.5$ ms with a repetition period of $T = 20$ ms (i.e., 50 Hz repetition rate). The double-pass AOM beam path included a polarizing beam splitter (PBS), quarter-wave plate (QWP), and a -75 mm concave mirror with an order-sorting iris to retroreflect the $+1$ order diffracted beam. The intensity-modulated light was coupled into a polarization-maintaining single-mode fiber and directed to the "Fiber input" of the optical setup shown in Fig. S18. Given the pulse duty cycle of 1/40, the average laser power reaching the microscope objective is 125 nW when the peak power (equivalently, the cw power) was measured to be 5 µW.

The resulting fluorescence collected at the "Fiber output" was routed through a separate free-space double-pass AOM setup for time gating. This collection AOM (Agiltron AOMS-115001111) was modulated by a TTL pulse of width $T_{\text{coll}} = 15$ ms after a waiting time $T_{\text{w}} = 50$ µs following the excitation pulse, as shown in the pulse sequence in Fig. S19. The 15 ms photodetection time results in a dead time T_{dead} of 4.45 ms in our measurements that suppressed the detection of background events, where the signal-to-noise ratio dropped below 1. The resulting excitation and gating sequence suppressed background arising from scattered laser light and enabled isolating Er^{3+} emission events in time. The temporally filtered fluorescence was coupled into a single-mode fiber and detected by a superconducting nanowire single-photon detector (SNSPD; PhotonSpot) operating at 770 mK with a timing jitter below 100 ps. Photon detection events were recorded for N pulse repetitions (corresponding to $\sim$ 13 s of integration) using a Swabian TimeTagger Ultra system (channel jitter $<$ 10 ps). The TimeTagger enabled time-gated photon counting by synchronously measuring photodetections within the gate-open time of the collection AOM.

S4.3 Lifetime Measurements

Lifetime measurements for all combinations of Er^{3+}-doped R-TiO_2 and A-TiO_2 films grown on GaAs and GaSb substrates are shown in Fig. S20. The same pulse sequence described in Sec. 4.2 was used for these measurements. The average laser power exciting the sample was between 120 nW (127 nW) for R-TiO_2 (A-TiO_2) samples. Measurements were performed at the excitation laser frequency of 197.167 THz (R-TiO_2) and 195.580 THz (A-TiO_2), corresponding to a frequency close to the $Z_1 \rightarrow Y_1$ transition peak. Although measurements were not taken exactly at the fitted center frequencies from the main text (Figs. 4 and 5), the extracted T_1 lifetimes showed negligible variation (within uncertainty) across several GHz near the center frequency, consistent with a broad inhomogeneous linewidth. Becuase the $Z_1 \rightarrow Y_1$ transition lifetime is longer in in R-TiO_2 than in A-TiO_2, integration time of 5 minutes ($N = 15000$) and 1 minutes ($N = 3000$) were used for R-TiO_2 (Figs. S20(a),(b)) and A-TiO_2 (Figs. S20(c),(d)), repsectively.

In each plot, the vertical dashed lines indicate the temporal region used for exponential fits of the form $A \times e^{-t/T_1}$, performed on datasets corrected for background detections and normalized by the maximum photodetections. Across both TiO_2 phases, Er^{3+} ions exhibited longer lifetimes in thin films grown on GaAs than on GaSb substrates, suggesting that substrate-induced strain or growth-related effects influence Er^{3+} incorporation within the TiO_2, which will be investigated in the future.

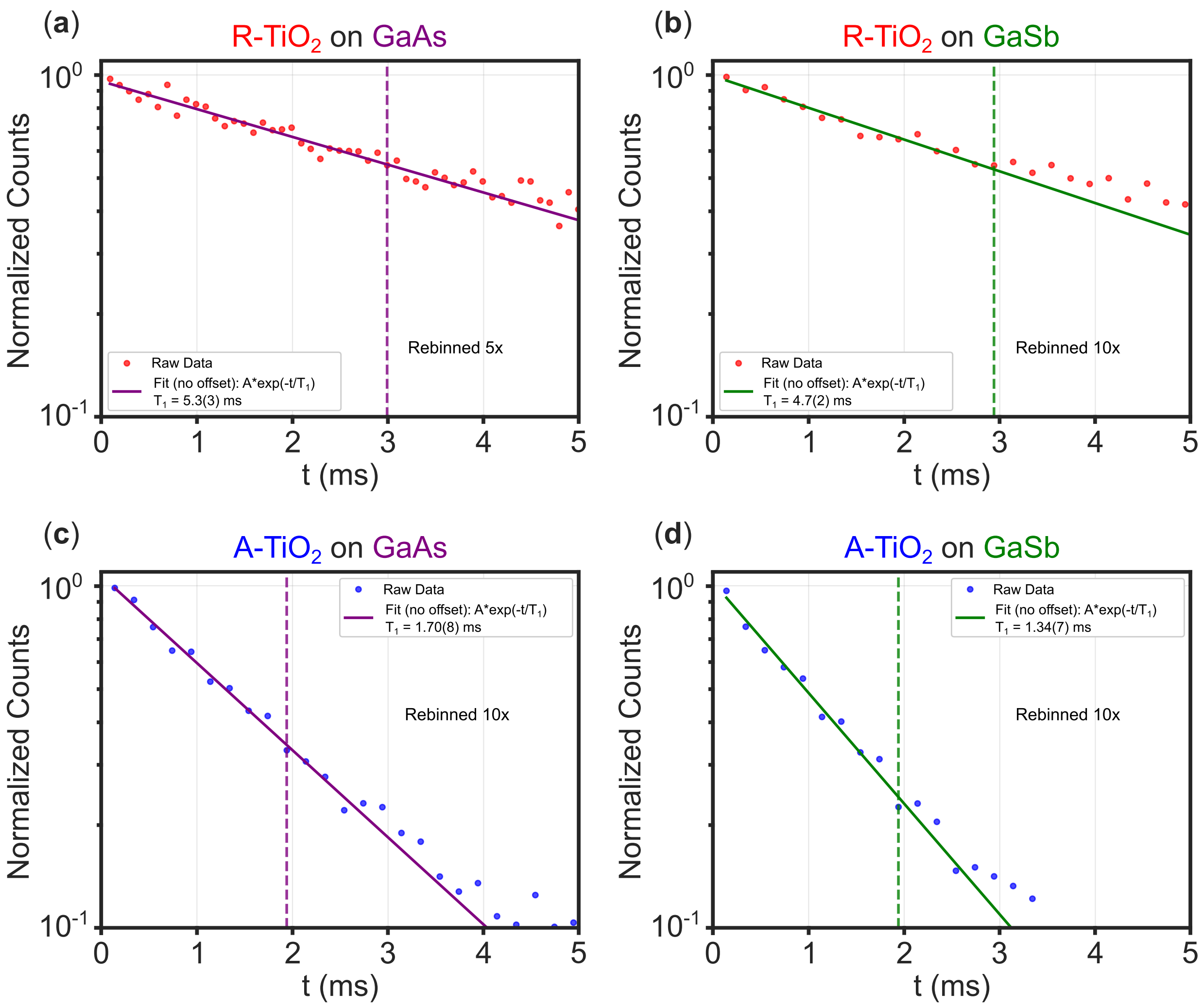

Figure S20: Normalized and background subtracted lifetime measurements from Er^{3+} $Z_1 \rightarrow Y_1$ optical transitions. Data were normalized after background subtraction and temporally rebinned to improve fit quality when signal counts were low. The rebinning employed in each dataset is indicated on the plot. (a) Results for sample GaAs-HT-4 (R-TiO_2 on GaAs). (b) Results for sample GaSb-HT-1 (R-TiO_2 on GaSb). (c) Results for sample GaAs-LT-1 (A-TiO_2 on GaAs). (d) Results for sample GaSb-LT-1 (A-TiO_2 on GaSb).

References

1. Lee, H. N.; Ambrose Seo, S. S.; Choi, W. S.; Rouleau, C. M. Sci. Rep. **2016**, 6, 19941.

2. Acevedo, W. R.; Aguirre, M. H.; Noheda, B.; Rubi, D. Oxygen vacancy engineering in pulsed laser deposited $BaSnO_3$ thin films on $SrTiO_3$. 2025; `https://arxiv.org/abs/2503.11957`.

3. Bell, C. N.; Lee, D.-C.; Drexler, M. N.; Rouleau, C. M.; Sasaki, K.; Senanayake, S. D.; Williams, M. D.; Alamgir, F. M. Thin Solid Films **2021**, 717, 138437.

4. Ji, C.; Solomon, M. T.; Grant, G. D.; Tanaka, K.; Hua, M.; Wen, J.; Seth, S. K.; Horn, C. P.; Masiulionis, I.; Singh, M. K.; Sullivan, S. E.; Heremans, F. J.; Awschalom, D. D.; Guha, S.; Dibos, A. M. ACS Nano **2024**, 18, 9929–9941.

5. Zur, A.; McGill, T. C. J. Appl. Phys. **1984**, 55, 378–386.

6. Cassels, J. W. S. An introduction to the geometry of numbers; Springer Science & Business Media, 2012.

7. Singh, M. K.; Grant, G. D.; Wolfowicz, G.; Wen, J.; Sullivan, S. E.; Prakash, A.; Dibos, A. M.; Joseph Heremans, F.; Awschalom, D. D.; Guha, S. J. Appl. Phys. **2024**, 136, 124402.

8. Parratt, L. G. Phys. Rev. **1954**, 95, 359–369.

9. Van Ommen, A. Appl. Surf. Sci. **1987**, 30, 244–264.

10. Wagner, S.; Povilonis, E. I. J. Electrochem. Soc. **1974**, 121, 1487.

11. Crank, J. The mathematics of diffusion; Oxford university press, 1979.

12. Atkinson, A. Rev. Mod. Phys. **1985**, 57, 437–470.

13. Cheng, H.; Selloni, A. Phys. Rev. B **2009**, 79, 092101.

14. Hairer, E.; Wanner, G. J. Comput. Appl. Math. **1999**, 111, 93–111.

15. Perez-Rodriguez, S.; Gonzalez-Pinto, S.; Sommeijer, B. J. Comput. Appl. Math. **2009**, 231, 49–66.
